# The Influence of POPC as a Coaggregate in Amyloid‑β Oligomer Formation

**DOI:** 10.1021/acschemneuro.5c00605

**Published:** 2025-09-16

**Authors:** Kelsie M. King, Emma M. Cleveland, Allison Pennington, Sarah Fuccello, Anne M. Brown

**Affiliations:** † Interdisciplinary Program in Genetics, Bioinformatics and Computational Biology, 1757 Virginia Polytechnic Institute and State University, Blacksburg, Virginia 24061, United States; ‡ Department of Systems Biology, Virginia Polytechnic Institute and State University, Blacksburg, Virginia 24061, United States; § Department of Biochemistry, Virginia Polytechnic Institute and State University, Blacksburg, Virginia 24061, United States; ∥ Research and Informatics, University Libraries, Virginia Polytechnic Institute and State University, Blacksburg, Virginia 24061, United States; ⊥ Virginia Tech Center for Drug Discovery, Virginia Polytechnic Institute and State University, Blacksburg, Virginia 24061, United States

**Keywords:** amyloid-β, 1-palmitoyl-2-oleoyl-*sn*-glycero-3-phosphocholine, molecular dynamics simulations, Alzheimer’s disease, amyloids

## Abstract

Alzheimer’s Disease (AD) progresses with the formation of neuronal plaques composed primarily of the 42-residue alloform of amyloid-β (Aβ_42_), whose oligomeric forms induce cytotoxicity by interacting with neuronal membranes, resulting in permeabilization and calcium ion leakage. In AD, elevated phospholipase activity disrupts lipid homeostasis and may increase the concentration of free lipids, such as 1-palmitoyl-2-oleoyl-*sn*-glycero-3-phosphocholine (POPC), in extracellular environments proximal to the membrane surface, potentially promoting Aβ_42_ insertion and toxicity. The coaggregation of Aβ_42_ with free lipids is believed to modulate mechanisms underlying Aβ_42_-induced cytotoxicity; however, these interactions are poorly understood. Molecular dynamics (MD) simulations were conducted to investigate Aβ_42_-POPC interactions and study the aggregation and structural morphologies of hexameric, octameric, and decameric Aβ_42_ in conjunction with free POPC in a 1:1 ratio. Clustering, radius of gyration, and eccentricity analyses revealed that POPC modulates Aβ_42_ oligomer morphology in a size-dependent manner. POPC increased compactness and sphericity in octameric and decameric systems, but had minimal or variable effects on hexamers. Hydrophobic interactions between Aβ_42_ and POPC hydrocarbon tails drove co-oligomerization, and increased hydrophobic solvent accessibility of Aβ_42_ peptides, altering the energetic profiles of hydrophobic and aromatic residues. To this effect, we hypothesize that Aβ_42_ coaggregation with POPC may nucleate additional oligomerization events through hydrophobic exposure of Aβ_42_. This work provides a mechanistic basis for early Aβ_42_ oligomerization events in lipid microenvironments, offering insights into neurodegenerative pathology.

## Graphical abstract

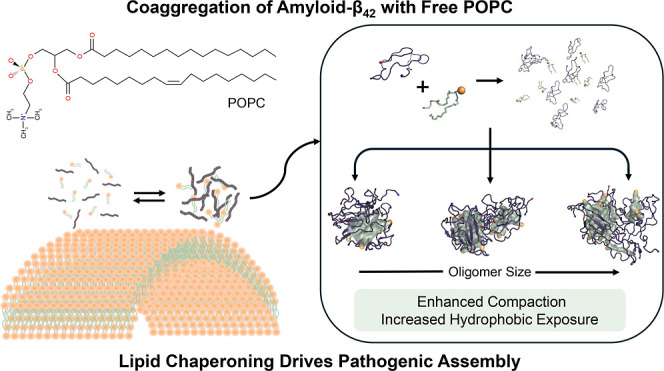

## Introduction

Alzheimer’s Disease (AD) poses a major public health threat as the leading form of dementia, affecting 50 million individuals worldwide.^1^ Often characterized by the physiological symptoms of cognitive decline, memory loss, and behavioral changes, the effects of AD also manifest as a substantial burden on patients, caregivers, and the economy.^2^ A notable signature of AD pathogenesis is the misfolding and aggregation of amyloid-β (Aβ), a 37–49 ^3^ residue peptide that exerts cytotoxic effects via accumulation in vasculature^4,5^ and membrane perturbations.^6–8^ A cleavage product of the transmembrane amyloid precursor protein (APP), the 40- and 42-residue variants are abundant in amyloid plaque depositions.^9,10^ Low-molecular-weight (LMW) Aβ oligomers are of particular importance for understanding AD disease-states, as oligomeric Aβ is thought to be the primary cytotoxic species.^11^ Aβ oligomers are transient and polymorphic;^12^ as such, the conformational dynamics of these species are highly sensitive to their aggregation environment.^13–20^ The effects of the lipid microenvironment on Aβ oligomerization dynamics are of particular interest, as Aβ is thought to exert cytotoxicity via membrane perturbations^8,21–26^ at the solvent–membrane interface. Membrane composition,^13–17^ and aqueous-phase lipids^27,28^ modulate amyloid conformational dynamics, and thus mechanisms of cytotoxicity. As such, it is of critical interest to investigate the effects of Aβ_42_ aggregation in biologically relevant microenvironments to gain atomistic-resolution insights into disease-related conformational states.

Interactions between amyloids and lipids, whether in equilibrium with membranes^27^ or as membrane components,^8,29^ often lead to membrane fluidity and loss of membrane integrity. Several models of Aβ-mediated membrane perturbations have been characterized, including pore,^30^ carpet,^31^ and detergent models,^8^ offering complementary hypotheses to explain Aβ-mediated membrane disruption (Figure 1A). The pore model suggests that oligomeric Aβ forms transmembrane channels or pores within the lipid bilayer, disrupting calcium ion homeostasis and inducing neuronal cell apoptosis.^6,32–34^ The detergent model describes Aβ-mediated membrane damage via lipid extraction and subsequent reorganization.^8,35,36^ The carpet model defines the mechanism of membrane binding and destabilization as employed by several antimicrobial peptide species, where membranes are permeabilized in response to widespread “carpeting” of peptides bound to the membrane.^37–41^ These mechanisms may not be mutually exclusive; two-step membrane disruption mechanisms have been observed that include pore formation and subsequent membrane fragmentation.^32^

**Figure 1:**
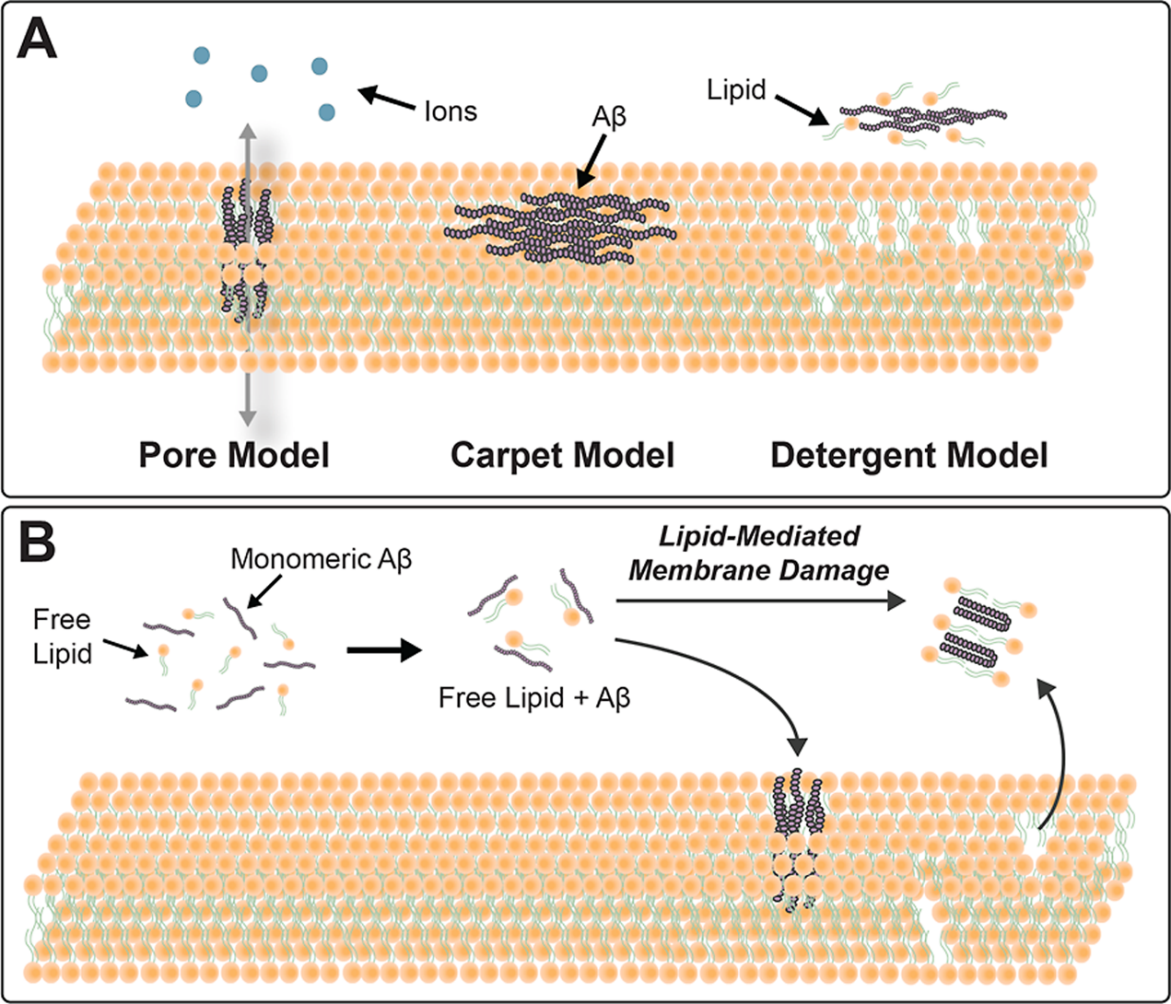
Mechanisms of amyloid-β induced membrane disruption. (A) Hypothesized mechanisms of membrane disruption by Aβ. The pore model (left) illustrates oligomeric Aβ forming ion channels or pores within the lipid bilayer, leading to ion leakage and membrane destabilization. The carpet model (middle) depicts Aβ peptides aligning parallel to the membrane surface, disrupting lipid packing and destabilizing the bilayer. The detergent model (right) demonstrates Aβ disintegrating the membrane into micelle-like structures through lipid extraction. (B) Proposed lipid-mediated perturbation of membranes by Aβ, described by Sciacca et al.^27^ Lipids at the membrane–solvent interface coaggregate with Aβ, which can mediate pore and detergent mechanisms.

Interestingly, free lipids in the aggregation environment have been found to modulate the mechanism of membrane perturbation observed by Aβ_40_ ^27,42^ and α-synuclein,^27^ the amyloidogenic peptide implicated in Parkinson’s disease progression.^43,44^ In the context of AD, elevated phospholipase activity has been shown to disrupt lipid homeostasis,^45^ potentially increasing concentrations of free lipids in extracellular spaces, and creating a microenvironment that promotes the insertion of Aβ_42_. While the products of phospholipase activity also include fatty acids, choline, and lysolipids, here we focus on phosphocholine (PC) lipids with double acyl chains. This choice reflects their abundance as major components of cell membranes and allows us to examine how membrane-derived lipids may participate in amyloid-lipid interactions. Double-chain PC lipids have previously been used in studies of intrinsically disordered protein interactions with membranes,^46^ including investigations of PC chain length modulating amyloid behavior.^27^ Sciacca et al.^27^ observe that free lipids present at critical micelle concentration (CMC), thus in equilibrium with their supramolecular structure, inhibit amyloid fibrilization and modify Aβ_40_-mediated membrane perturbations in a phospholipid acyl chain length dependent manner (Figure 1B). Using varying chain lengths of PC lipids (22:1, 20:1, 18:1, 16:1, 14:1), larger acyl chains were found to favor a detergent-like mechanism. In contrast, shorter-chain lipids favored pore formation as determined through dye leakage assays. The authors suggest that the formation of amyloid-lipid complexes promotes membrane insertion via lipid-mediated protein transport. This phenomenon has been observed for the heat shock protein Hsc70, where free lipids bind to assist folding and membrane translocation for pore formation,^47^ supporting the notion that a lipid-mediated mechanism of membrane insertion could extend to amyloid oligomer models.^45^

The current understanding of AD emphasizes the critical role of Aβ peptide oligomerization in its pathogenesis. However, the influence of lipid coaggregation with Aβ peptides on oligomer conformational dynamics remains largely unexplored. Specifically, understanding the dynamics of Aβ_42_-lipid coaggregation and subsequent interactions with membranes is important for advancing our understanding of Aβ-related disease states. Computational methods, like molecular dynamics (MD) simulations, are powerful tools for probing the atomistic conformational landscape of transient species difficult to isolate in vitro.^48,49^ The wide availability of lipid parameters has enabled the computational study of Aβ oligomerization in various membrane environments, highlighting the dependence of oligomeric structure on membrane composition.^13,50–52^ Monomeric Aβ_40_ and Aβ_42_ have been simulated in complex with 22:1 and 14:1 PC molecules, indicating that hydrophobic interactions with lipid acyl chains are predominant in Aβ-lipid complexes.^27^ At the time of writing, no computational experiments have been performed to explore the oligomerization of Aβ_42_ with free lipids. Furthermore, there has yet to be a detailed computational investigation that compares the properties of multiple oligomer sizes for full-length Aβ_42_.

Here, we utilize all-atom MD simulations to observe the oligomerization of hexameric, octameric, and decameric Aβ_42_ in a 1:1 ratio with free 1-palmitoyl-2-oleoyl-*sn*-glycero-3-phosphocholine (16:0–18:1 POPC) in solvent. A 1:1 ratio was chosen to adequately sample Aβ_42_-POPC interactions with an optimal computational overhead. POPC was chosen due to the use of PC lipids in experimental work probing Aβ-lipid interactions,^27^ and its abundance in biological membrane environments.^53^ Oligomerization of hexameric, octameric, and decameric Aβ_42_ without free POPC was simulated as control. We observe that POPC acts as a chaperone to coordinate the formation of highly compact oligomers in a size-dependent manner. This work provides a mechanistic basis for phenomena observed in experimental studies, elucidating the role of Aβ_42_ microenvironment on oligomer dynamics and function.

## Results & Discussion

### Oligomer Size Influences Degree of Structural Compaction in Aβ_42_ Controls

Several low-molecular-weight (LMW) Aβ oligomer species have been resolved via NMR and ion-mobility mass spectrometry; tetrameric,^30^ hexameric,^54^ and octameric^30,55^ species are particularly prevalent. Furthermore, β-barrels composed of macrocyclic derivations of Aβ_16–36_ have been resolved with X-ray crystallography.^56,57^ Decameric Aβ species are not well-documented in the literature, although decameric species cross-seeded with amylin have been identified.^58^ Given the incidence of particular LMW-oligomer sizes, we simulated hexameric, octameric, and decameric Aβ_42_ oligomer formation to probe their oligomeric architecture and aggregation pathway. An initial morphological characterization of simulated Aβ_42_ oligomer systems was performed by examining RMSD cluster structures for every 200 ns of simulation time (Figures 2, and S1–S3). Control oligomers of all sizes appear to be relatively disordered, sampling predominantly random coil; secondary structure calculations confirmed this observation, with oligomers sampling ∼74–84% random coil structure across all replicates and systems (Figure S4). β-strand content was primarily sampled by residues ^17^LVFFA,^21^ ^32^ILGM,^35^ and ^38^GVV,^40^ forming a 2–3 stranded β-sheet, comprising ∼19–20% of sampled secondary structure content for controls. This hydrophobic β-strand motif is consistent with secondary structure data from native ion mobility mass spectrometry experiments.^54,59^ Transient helical content was also sampled, where residues ^12^VHH^14^ were the most likely to adopt helical content (2 ± 0.1% across all replicates and systems), followed by ^23^NVGSQK^28^ (1 ± 0.1% across all replicates and systems).

**Figure 2:**
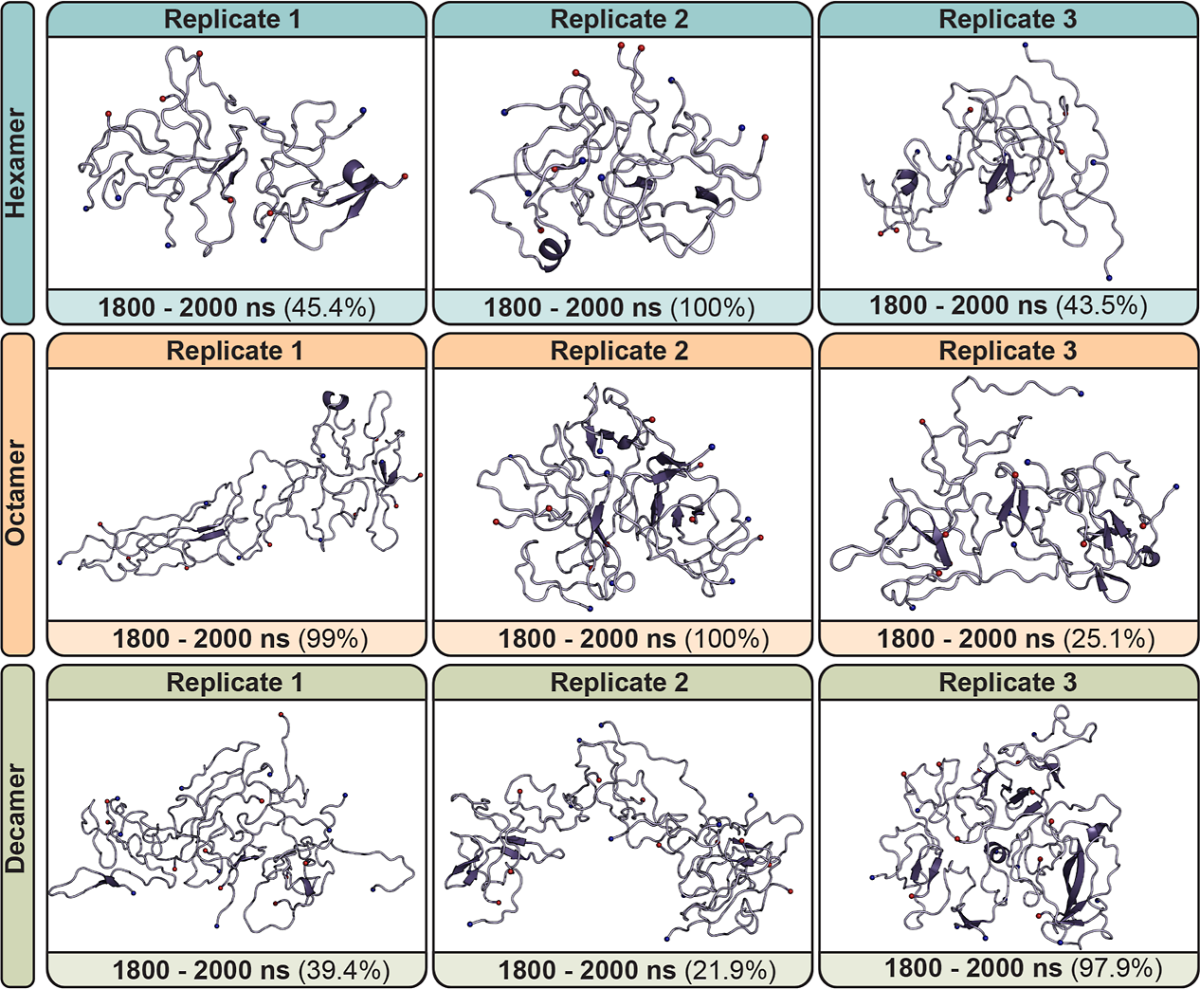
Dominant morphologies from RMSD clustering of hexameric, octameric, and decameric Aβ_42_ controls. For each replicate, clustering was performed on backbone atoms with a 0.3 nm cutoff. Clusters here represent the dominant morphology obtained over the last 200 ns of simulation (1800–2000 ns). Percentages indicate the percentage of frames within 0.3 nm of the presented structure over the clustering time frame. Aβ_42_ is shown as a purple cartoon. The N- and C-termini are shown as blue and red spheres, respectively.

Visual inspection of clusters over time indicates size-dependence with respect to oligomerization state. To probe how oligomerization dynamics are impacted by oligomer size, minimum distance between Aβ_42_ peptides over time were calculated (Figure S5). Hexameric Aβ_42_ aggregated fastest, followed by octameric and decameric Aβ_42_. When examining the populations of *N*-mer sizes sampled during aggregation, it is observed that each system populates smaller, transient oligomer species on the pathway to stable hexamer, octamer, or decamer formation (Figures S6–S8). Thus, the apparent positive relationship between oligomer size and aggregation time may be attributed to more possible intermediate states in larger oligomer systems, thereby increasing the kinetic barrier to aggregation. This observation is supported by radius of gyration (*R*_g_) calculations, which indicates a positive relationship between oligomer size and compactness (Figure S9, Table 1), where hexameric *R*_g_ (mean = 2.1 ± 0.5 nm) < octameric *R*_g_ (mean = 2.6 ± 0.3 nm) < decameric *R*_g_ (mean = 2.9 ± 0.3 nm) (*p* < 0.05). In tandem with *R*_g_, eccentricity calculations reveal a linear relationship between oligomer size and shape (Figure S10, Table 1). Hexameric Aβ_42_ on average adopts more spherical conformations (mean = 0.87 ± 0.07), followed by octameric (mean = 0.89 ± 0.11) and decameric Aβ_42_ (mean = 0.92 ± 0.04), which progressively adopt more elongated structures. Hexameric Aβ_42_ species observed with small angle neutron scattering are reported to adopt *R*_g_ values between 2 and 4 nm,^60^ consistent with our computational results. Spherical aggregates are one of several commonly observed Aβ_42_ morphologies in experimental work.^61–63^ Solid-state NMR has identified a spherical aggregate with a disordered N-terminus and hydrophobic β-strand content,^64^ consistent with the sampled morphologies in our simulations. Additionally, spherical aggregates with secondary structure characteristics sampled in this work have been sampled computationally with other force fields.^65^

**Table 1:** Radius of Gyration (*R*_g_) and Eccentricity for Aβ_42_ Control Oligomers^a^

	Aβ_42_ hexamer control		Aβ_42_ octamer control		Aβ_42_ decamer control	
replicate	*R*_g_ (nm)	eccentricity	*R*_g_ (nm)	eccentricity	*R*_g_ (nm)	eccentricity
1	2.3 ± 0.1	0.91 ± 0.02	3.2 ± 0.1	0.97 ± 0.01	2.8 ± 0.1	0.93 ± 0.02
2	2.2 ± 0.4	0.82 ± 0.09	2.3 ± 0.2	0.76 ± 0.09	3.2 ± 0.5	0.93 ± 0.03
3	2.2 ± 0.1	0.89 ± 0.03	2.9 ± 0.2	0.93 ± 0.05	3.1 ± 0.6	0.91 ± 0.04
average	**2.3** ± **0.3**	**0.87** ± **0.07**	**2.8** ± **0.4**	**0.89** ± **0.11**	**3.0** ± **0.5**	**0.92** ± **0.03**

a Average taken from 0.5–2 μs of the simulation period, corresponding to the period at which stable oligomers were formed for all systems (see Figures S6–S8). Note that eccentricity averages and uncertainties are reported with two significant digits as more precision is required to accurately convey system properties.

Analysis of *R*_g_, eccentricity and clustering data indicate that smaller oligomeric species aggregate faster, generally adopting more compact structures relative to their larger counterparts. It should be noted, however, that there was significant variation in compaction across replicates (*p* < 0.05), implying that compaction is somewhat stochastic, or that compaction is dependent on intermolecular interactions present. Furthermore, increased simulation time or enhanced sampling techniques to sample a larger conformational space may be necessary to say this trend is definitively true. Together, aggregation analysis, *R*_g_, and eccentricity indicate that smaller oligomeric species aggregate faster, generally adopting more spherical and compact structures relative to their larger counterparts. This may contribute to the higher incidence of smaller-sized LMW-oligomers (dimeric-octameric) in the literature.^30,54–57^

### POPC Promotes Aβ_42_ Oligomer Compaction via Hydrophobic Interactions

Experimental studies have indicated that coaggregation of Aβ with lipids significantly alters fibrilization rates and modulates mechanisms of model bilayer perturbations.^27^ However, a detailed structural and mechanistic characterization with respect to lipid aggregation with LMW-oligomeric species does not currently exist. As PC lipids are ubiquitous and abundant in lipid bilayers,^53,66^ we simulated Aβ_42_ hexamer, octamer, and decamer formation in 1:1 ratio with 16:0–18:1 POPC. To probe the structural characteristics of Aβ_42_/POPC oligomers, clustering and secondary structure calculations were performed (Figures 3, and S11–S14). Interestingly, Aβ_42_/POPC oligomers of all sizes largely retained the secondary structure features present in the control simulations. The hydrophobic β-sheet motif comprised of segments ^17^LVFFA,^21^ ^32^ILGM,^35^ and ^38^GVV^40^ were retained; overall, β-strand content was not significantly changed. There was a marginal enrichment in β-strand content for hexameric (19 ± 5% vs 21 ± 4%, control vs POPC) and octameric systems (20 ± 4% vs 22 ± 4%, control vs POPC), but slightly decreased in decamer systems (19 ± 3% vs 18 ± 3%, control vs POPC). Like controls, the transient ^12^VHH^14^ helix was also sampled in all Aβ_42_/POPC oligomer sizes (Figure S14).

**Figure 3:**
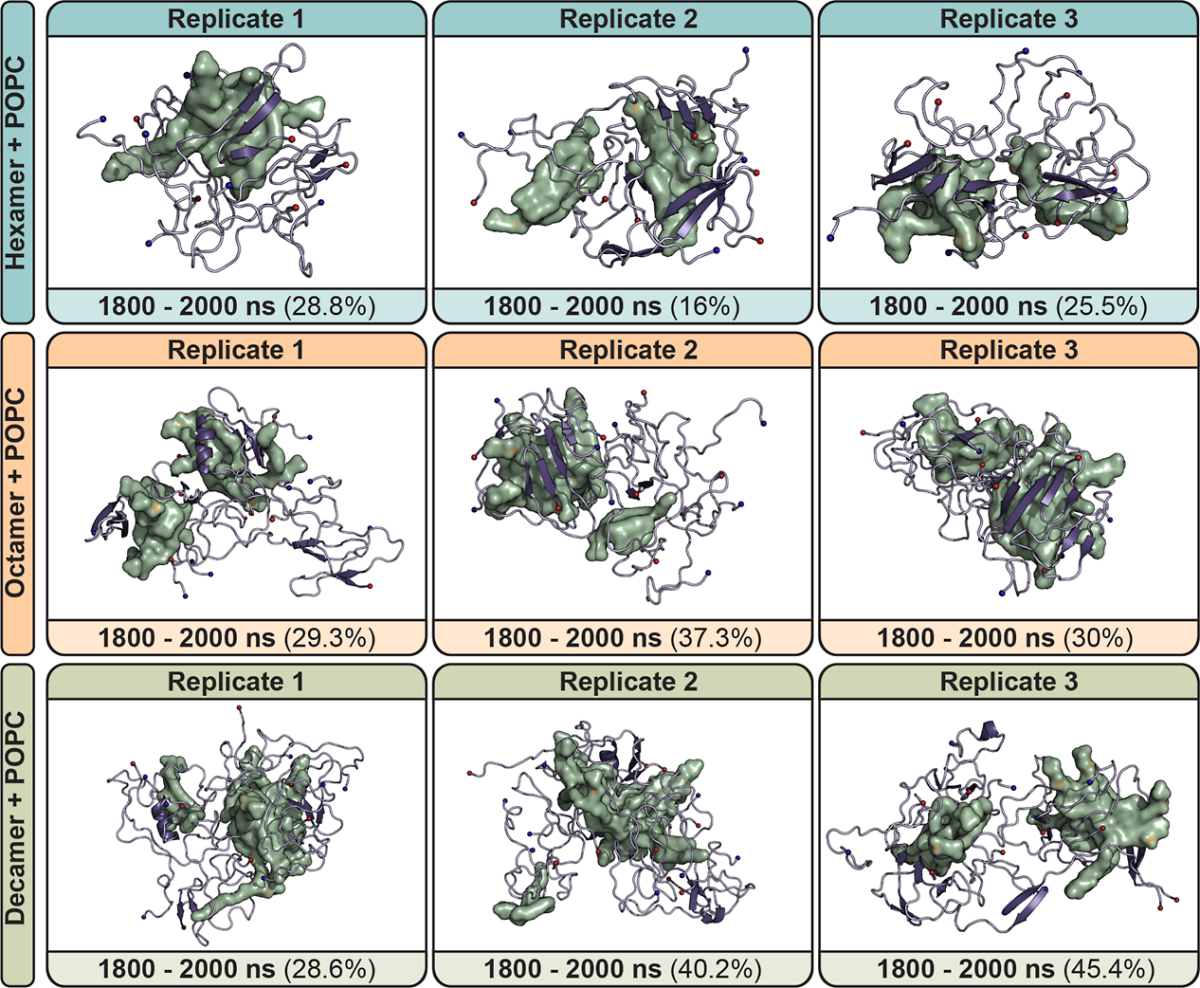
Dominant morphologies from RMSD clustering of hexameric, octameric, and decameric Aβ_42_ with POPC. For each replicate, clustering was performed on protein backbone atoms with a 0.3 nm cutoff. Clusters here represent the dominant morphology obtained over the last 200 ns of simulation (1800–2000 ns). Percentages indicate the percentage of frames within 0.3 nm of the presented structure over the clustering time frame. The aggregation time is the amount of simulation time taken to form a stable, nondissociating oligomer structure as based on aggregation analysis (see Figures S6–S8) Aβ_42_ is shown as a purple cartoon. The N- and C-termini are shown as blue and red spheres, respectively. POPC is shown as a green surface.

In control oligomer systems, a positive relationship between oligomer size and aggregation speed was observed. In Aβ_42_/POPC oligomer simulations, this relationship is not as well-defined as the aggregation speed of Aβ_42_ peptides varied considerably between replicates of the same oligomer size when coaggregated with POPC (Figures S6–S8). The range of sampled time scales for the formation of higher-order oligomers are considerably wider for hexameric (71–127 ns vs 39–253 ns, control vs POPC) and octameric (191–273 ns vs 445–1400 ns, control vs POPC) species. Formation of decameric Aβ_42_/POPC was generally achieved within a similar or smaller time scale relative to controls (372–1254 ns vs 147–538 ns, control vs POPC), indicating that POPC accelerated decamer formation, but not hexamer or octamer formation. Despite the apparent variability in aggregation kinetics, octameric and decameric Aβ_42_/POPC oligomers were significantly more compact than their control counterparts (*p* < 0.05) (Table 2, Figure S9). Furthermore, hexameric and decameric oligomers were more spherical in shape when simulated with POPC relative to controls, as exhibited by eccentricity calculations (Table 2, Figure S10). The increased compaction and increased aggregation rate exhibited in decameric Aβ_42_/POPC simulations suggests that lipids in the oligomerization microenvironment may act as molecular chaperones for oligomerization events, in agreement with experimental observations.^27^ The chaperoning effect is particularly evident for larger oligomer sizes (octamer and decamer), where POPC may act to decrease kinetic barriers associated with their aggregation. Experimental work with free POPC and free POPG indicate that the presence of free lipids accelerate nucleation events in the aggregation of both lysozyme and Aβ_42_,^67^ in agreement with the observations in this work.

**Table 2:** Radius of Gyration (*R*_g_) and Eccentricity for Aβ_42_/POPC Oligomers^a^

	Aβ_42_/POPC hexamer		Aβ_42_/POPC octamer		Aβ_42_/POPC decamer	
replicate	*R*_g_ (nm)	eccentricity	*R*_g_ (nm)	eccentricity	*R*_g_ (nm)	eccentricity
1	2.0 ± 0.1	0.66 ± 0.08	2.8 ± 0.4	0.90 ± 0.03	2.5 ± 0.1	0.80 ± 0.04
2	2.1 ± 0.1	0.83 ± 0.03	2.5 ± 0.1	0.88 ± 0.02	2.6 ± 0.1	0.89 ± 0.03
3	2.2 ± 0.04	0.82 ± 0.02	2.9 ± 0.7	0.91 ± 0.04	3.0 ± 0.2	0.92 ± 0.02
average	2.1 ± 0.1	0.77 ± 0.08	2.8 ± 0.5	0.90 ± 0.04	2.8 ± 0.3	0.87 ± 0.06
control average	2.3 ± 0.3	0.87 ± 0.07	2.8 ± 0.4	0.89 ± 0.1	3.0 ± 0.5	0.92 ± 0.03

a Average taken from 0.5–2 μs of the simulation period, corresponding to the period at which stable oligomers were formed for all systems (see Figures S6–S8). Averages for control oligomers of the same size are listed below Aβ_42_/POPC system averages. Note that eccentricity averages and uncertainties are reported with two significant digits as more precision is required to accurately convey system properties.

The polar head of POPC is zwitterionic, containing both a positively charged choline group and a negatively charged phosphate group, attached to the hydrophobic tails via a glycerol linker (Figure 4A). NMR experiments of Aβ_40_ incubated with 14:0 PC indicate that peptide-lipid interactions are mediated initially via electrostatic interactions in the Aβ_40_ N-terminus, followed by a structural rearrangement where hydrophobic interactions were enriched.^27^ To probe the primary residue regions involved in POPC binding, interaction frequencies were calculated between individual residues and lipid choline, phosphate, glycerol, and tails (Figure 4B). In all oligomer sizes, Arg5 and Tyr10 were the most likely to interact with lipid polar regions. There is a bimodal distribution of residue interactions with the POPC lipid tails; residues ^17^LVFF^20^ and ^32^IGLMV^36^ exhibit the highest interaction propensities with lipid tails. To decipher the mechanism of Aβ_42_/POPC binding, minimum distances between two groups were calculated: (1) Aβ_42_ hydrophobic residues (Ala, Val, Leu, Ile, Met, Phe) and POPC hydrocarbon tails, and (2) Aβ_42_ polar/charged residues (Asp, Glu, Arg, Lys, Ser, Thr, Asn, Gln) and the polar POPC head (Figure S15). In all systems and replicates, hydrophobic residues rapidly interact with POPC tails, converging to a minimum distance of ∼0.3 nm in the first ∼5–10 ns of simulation. Polar and charged residues take longer to converge to a minimum distance within the threshold for salt bridges (0.4 nm) or hydrogen bonds (0.35 nm), suggesting that Aβ_42_-POPC binding is initially mediated by hydrophobic interactions. Computing minimum distance for all residues (regardless of side chain property) between POPC heads and tails indicates that overall, interactions with the POPC headgroup are marginally slower to converge than interactions with hydrophobic tails; however, convergence is reached faster than when filtering based on residue side chain property (Figure S16). This suggests that backbone hydrogen bonding between hydrophobic regions and the POPC polar head may be a mediator of interaction in some replicates. Hydrogen bond analysis indicates that, in addition to N-terminal residues, Gly25, Ser26, Lys28, Gly33, Leu34, and Gly37 exhibit a propensity to hydrogen bond with POPC (Figure S17). While our simulations indicate that glycine residue backbones are common hydrogen bond partners with POPC, previous simulations of a preformed Aβ_42_ tetramer with an SDS molecule indicate that Gly33, Gly37 and Gly38 are critical to SDS-Aβ_42_ interaction via interaction with the SDS hydrocarbon tail.^25^ Previous simulations using the polarizable Drude oscillator indicate that glycine residues in amyloid fibrils^68^ and oligomers^69^ are electronically plastic, making glycine amenable to acting as a polar or hydrophobic residue depending on microenvironment, which could explain this discrepancy. Together, these data indicate that POPC binding is mediated by both hydrophobic and electrostatic interaction between POPC and the hydrophobic core/C-terminal of Aβ_42_.

**Figure 4:**
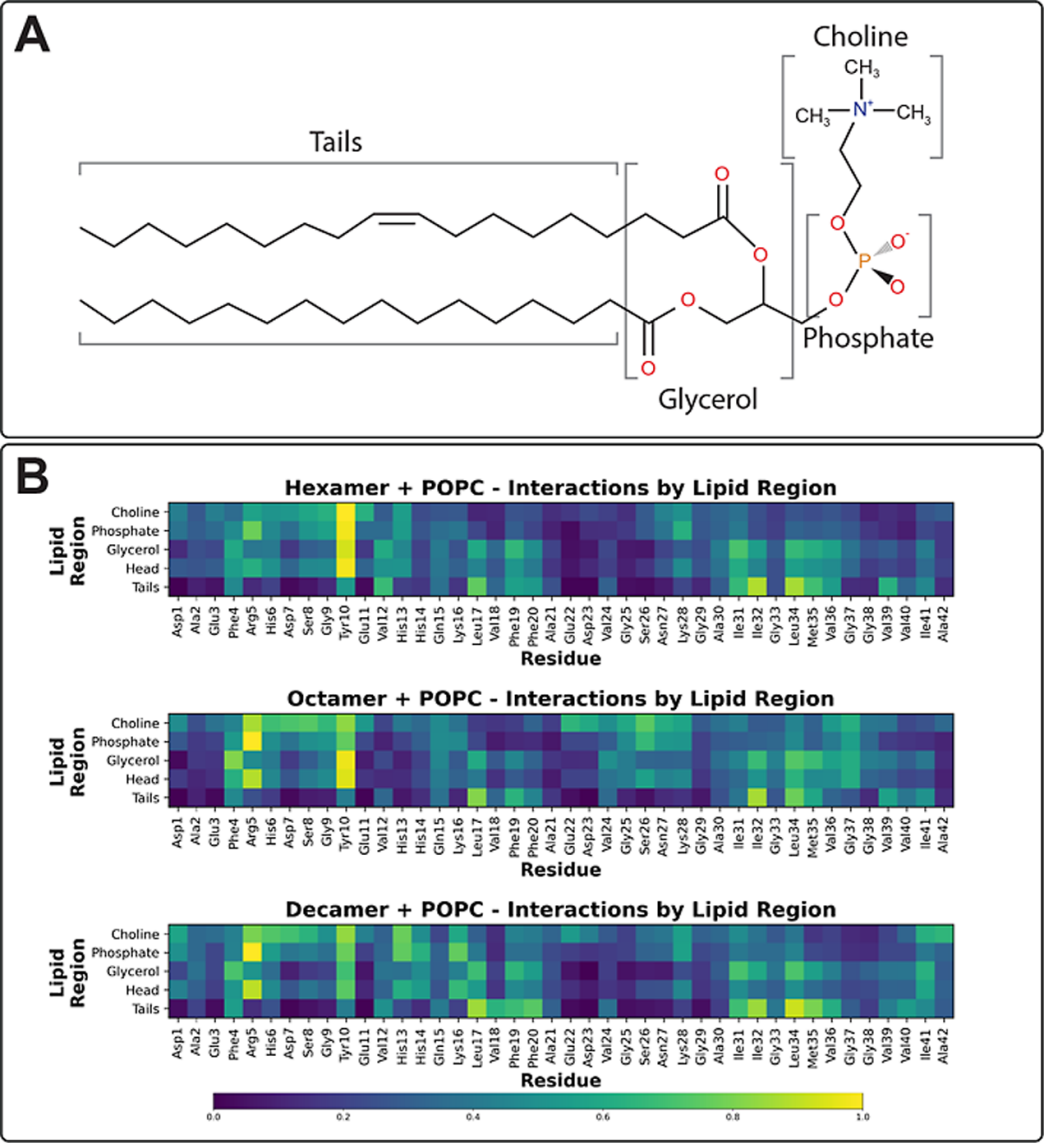
2D-structure of POPC and residue interactions by POPC region. (A) 2D-structure of POPC, with brackets labeling regions used in residue distance calculations. (B) Interaction probability heatmaps of POPC-residue interactions by POPC region, for hexameric, octameric, and decameric Aβ_42_.

### POPC Alters Aβ_42_ Thermodynamic Stability via Hydrophobic Exposure

The current body of literature, both experimental and theoretical, indicates that Aβ_42_ oligomerization is primarily driven by the burial of hydrophobic regions in aqueous environments.^70,71^ Inter-residue interaction heatmaps indicate that interactions between hydrophobic residues, like the hydrophobic core (^17^LVFFA^21^), and C-terminus (^30^GAIIGLMVGGVVIA^42^), are ubiquitously sampled in control oligomer simulations (Figure S18). Furthermore, residue solvent-accessible surface area (SASA) correlates well with binding free energy decompositions in control simulations, indicating that oligomerization events in control simulations are driven by hydrophobic occlusion from solvent (Figure S19). However, in the presence of POPC, the hydrophobic SASA of Aβ_42_ peptides is significantly increased with respect to the controls for all oligomer sizes, suggesting that POPC remodels traditional oligomerization mechanisms (Figure 5A). This is highlighted by a loss of interaction density between hydrophobic C-terminal residues in all Aβ_42_/POPC oligomers relative to controls (Figure S18). Visualizing dominant morphologies from simulations indicates that hydrophobic β-strand motifs organize themselves along the surface of POPC hydrocarbon chains, where the POPC chains appear to be internal to the oligomer (Figure 5B), forcing residues otherwise involved in control oligomer internal packing to the aggregate surface. Indeed, analysis of interactions with solvent indicates that POPC exhibits, on average, much lower levels of interaction with solvent relative to Aβ_42_ peptides when normalized for molecule size (Figure S20). This indicates that POPC hydrocarbon tails are preferentially buried.

**Figure 5:**
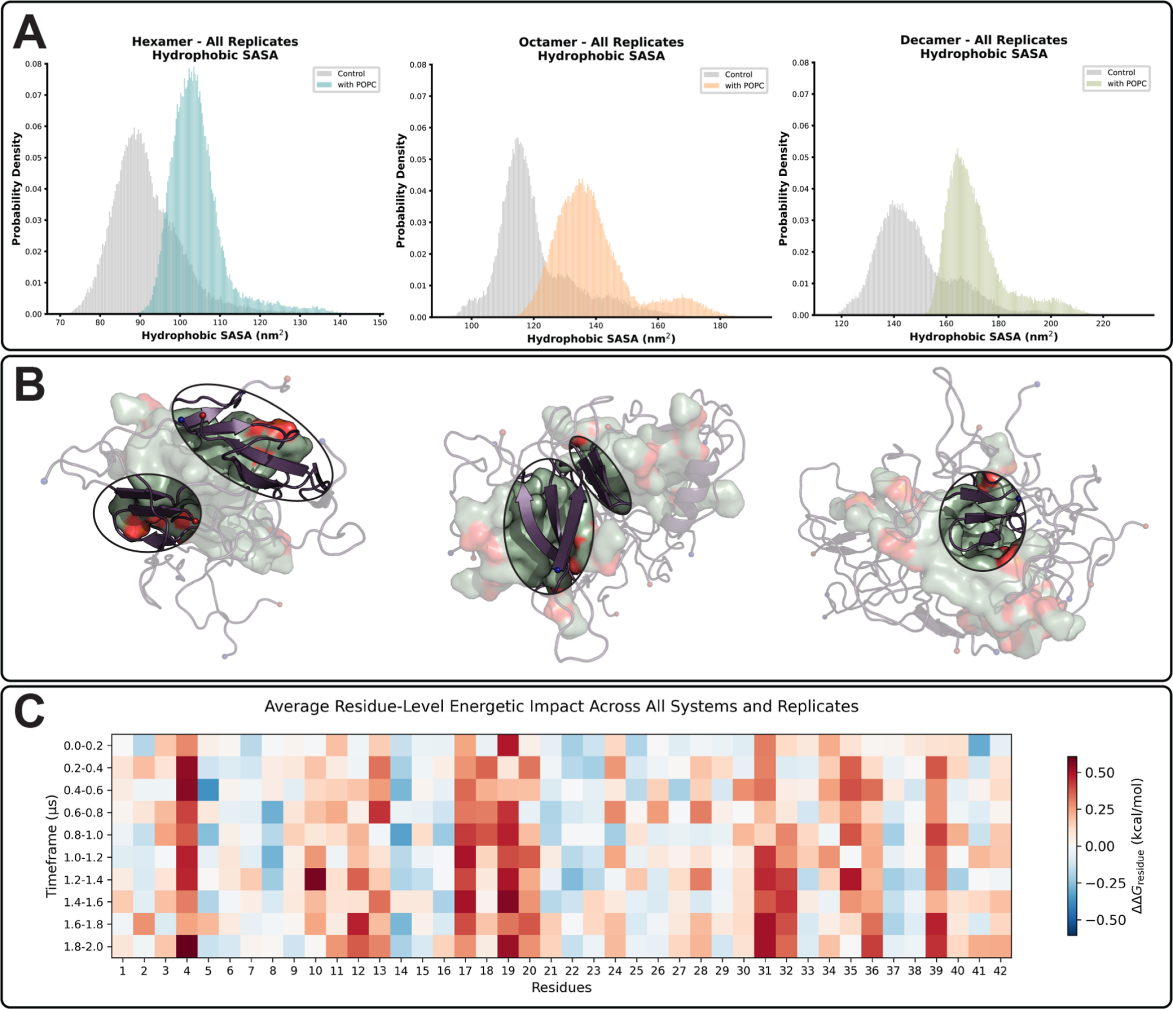
Hydrophobic solvent accessible surface area is increased in Aβ_42_/POPC oligomers relative to controls, altering residue energetic profiles. (A) Hydrophobic solvent accessible surface area distributions. Gray distribution represents Aβ_42_ control oligomers, and colored distribution represents Aβ_42_/POPC oligomers. (B) Snapshots from simulation highlighting β-strands comprised of hydrophobic residues along POPC surface. POPC shown as surface. Green corresponds to carbon atoms, red/orange corresponds to oxygens and phosphates, respectively. Aβ_42_ shown as purple cartoon. N- and C-termini are colored as red and blue spheres, respectively. (C) Heatmap displays per-residue energy differences between control and POPC-exposed systems derived from FoldX^72,73^ analysis of clustered simulation data. Due to the absence of statistically significant differences between oligomer sizes, data from all replicates and systems were averaged by residue for each 200 ns time frame. The *y*-axis represents time in 200 ns intervals, while the *x*-axis denotes Aβ_42_ residue number. Red indicates higher Δ*G* values in the POPC system compared to control, blue indicates lower Δ*G*, and white represents no difference between conditions.

Given the apparent remodeling of oligomerization mechanisms, we examined changes to the thermodynamic stability of Aβ_42_ oligomers in the presence of POPC.^74,75^ To investigate the influence of free lipids on the energetics of Aβ_42_ oligomers, we performed a per-residue energy decomposition analysis using FoldX.^72,73^ The heatmap of ΔΔ*G* (Δ*G*_POPC_ – Δ*G*_Control_) across residues and time indicates that the core and C-terminal regions, including residues 4, 10–13, 17–20, and 31–40, were less energetically favorable in aggregates with POPC (Figure 5C). Residue-level analysis revealed that aromatic and hydrophobic residues are the most strongly affected by POPC, with significant Δ*G* differences between control and POPC-exposed systems across all oligomer sizes (*F* = 12.5, *p* = 3.7 × 10^–12^ for aromatics; F = 18.1, *p* = 5.7 × 10^–18^ for hydrophobics; Table 3). Here, the F-statistic from one-way ANOVA represents the ratio of variance between groups, defined by condition and oligomer system, to variance within replicates. Larger *F*-values indicate stronger effects of POPC relative to control. Charged and polar residues showed no significant change (*p* > 0.05). For hydrophobic and aromatic residues, particularly Phe, Tyr, Val, Leu, and Ile residues, the increase in ΔΔ*G* is positively correlated with an increase in solvent accessibility when co-oligomerized with POPC (*R* ^2^ = ∼0.44–0.69), providing a mechanistic explanation for the changes to thermodynamic stability (Figure S21).

**Table 3:** ANOVA Results Assessing Δ*G* Differences between Control and POPC-Exposed Systems by Residue Type^a^

Residue type	ANOVA (F, p)	significant Comparisons (FDR-adj *p* < 0.05)	key findings
aromatic	*F* = 12.5, *p* = 3.7 × 10^–12^	control vs POPC (all sizes)	strong POPC effects, strongest in decamers. no size dependence
hydrophobic	*F* = 18.1, *p* = 5.7 × 10^–18^	control vs POPC (all sizes)	extremely strong POPC effects across all systems. no size dependence
charged	*F* = 1.5, *p* = 0.18	none	no significant effects of POPC or system size
polar	*F* = 0.7, *p* = 0.63	none	no significant effects of POPC or system size

a One-way ANOVA was used to compare per-residue decomposition free energy (Δ*G*) across systems with and without POPC for each residue type. *F*-statistics quantify the ratio of variance between groups to variance within replicates. *P*-values report the significance of these differences. Aromatic and hydrophobic residues exhibited statistically significant differences (FDR-adjusted *p* < 0.05), with the most substantial POPC effects observed in decamer systems.

The residue regions that exhibited the largest shift toward thermodynamic instability were those with the highest probability of adopting secondary structure—particularly β-sheets—which were slightly, but significantly enriched in simulations with POPC (*p* < 0.05) (Figure S14). The formation of hydrophobic β-sheets is critical to the stability and formation of globular and fibrillar amyloid assemblies alike.^74,76,77^ As such, the apparent destabilization of these β-strand regions as sampled in our simulations is contradictory to the body of literature. Based on these results, we propose a few explanations. First, the solvent exposure of POPC hydrocarbon tails is highly unfavorable, as lipids assemble into monolayers/bilayers/micelles in aqueous environments. It is hypothesized that the thermodynamic instability of free lipids in an aqueous environment is the primary driver of Aβ_42_-POPC aggregation in the context of our simulations; this is supported by the numerous hydrophobic contacts between hydrophobic Aβ_42_ regions and POPC hydrocarbon tails, which bury POPC tails internally to the oligomer structure (Figures 4B, and S20). Second, hydrophobicity is associated with faster aggregation kinetics and stability of fibril assemblies.^71,78,79^ We suggest that the thermodynamic instability of exposed hydrophobic β-strand regions could serve as a nucleator for further assembly events driven by the occlusion of these regions from solvent.

Together, these observations lead us to hypothesize that free 16:0–18:1 POPC in the aggregation environment may enable the formation of higher-order Aβ_42_ oligomers by providing a scaffold for initial oligomerization events. Dominant morphologies of Aβ_42_ co-oligomerized with POPC suggest the formation of lipid clusters to which Aβ_42_ peptides bind to, enriching the formation of hydrophobic β-strand motifs. β-strand motifs comprised of hydrophobic Aβ residues are known to act as nuclei in fibril polymerization events.^80,81^ The increased hydrophobic surface area in Aβ_42_/POPC oligomers could potentially provide a surface for secondary nucleation events, contributing to amyloidogenic cascades involved in disease states.

## Conclusions

This work examines the role of free POPC in modulation of Aβ_42_ oligomerization dynamics. We simulated the formation of hexameric, octameric, and decameric Aβ_42_, both with and without POPC, to understand the effects on oligomers of various sizes. POPC acted as a chaperone in aggregation events, particularly for octameric and decameric Aβ_42_, resulting in oligomers with significantly increased compaction (*p* < 0.05) with respect to controls. The chaperone phenomenon observed here is mediated by both hydrophobic and electrostatic interactions, but interactions with aromatic and hydrophobic residues, like Phe4, Phe19, and Phe20, are more widespread and probable. Hydrophobic β-strands formed across the hydrocarbon surface of POPC chains, exposing these regions to solvent, potentially creating a nucleation surface for further aggregation events. This work provides the first detailed view into the dynamics of Aβ_42_ oligomer formation in the presence of free lipids, which are hypothesized to drastically alter Aβ_42_ aggregation dynamics and mechanisms of cytotoxicity. This work also provides a framework for investigations into Aβ_42_ interactions with other lipids, and investigations into surfactant-modulated Aβ_42_ interaction with cellular membranes.

## Methods

All MD simulations were constructed with and run using the GROMACS^82,83^ software package, versions 2020.4. The CHARMM36m (C36m) force field^84,85^ was employed for all simulations. C36m is optimized for the simulation of intrinsically disordered proteins,^86^ and has parameters available for several lipids, including POPC.^87^ All systems employed the TIP3P water model,^88^ in 150 mM KCl at 310 K.

### Oligomeric System Construction and Simulation

Prior to simulation of oligomers, a single Aβ_42_ monomer was simulated for 1000 ns to generate a starting peptide structure in solution. The system was built with CHARMM-GUI^89^ and PDB ID: 1IYT^90^ was utilized. The peptide used in subsequent simulations was the dominant structural morphology from RMSD clustering^91^ over the last 500 ns of the monomer simulation. Six systems were simulated: hexamer control, octamer control, decamer control, hexamer + POPC, octamer + POPC, and decamer + POPC. POPC parameters and structure were utilized from CHARMM-GUI.^92^ Aβ_42_ systems with POPC were simulated in a 1:1 Aβ_42_/POPC ratio (e.g., in hexamer system, there are 6 Aβ_42_ peptides and 6 POPC molecules). Each system was simulated in triplicate. Peptides and/or POPC chains were placed such that they were 1.0+ nm apart at the time of construction (see Table 4.1 for box sizes). Energy minimization was performed using the steepest descent method. NVT, and subsequently *NPT* ensembles were applied for equilibration, using the Nosé-Hoover^93^ thermostat and the Parinello-Rahman^94^ barostat, using 310 K and 1 bar as the reference temperature and pressure, respectively. Position restraints on heavy atoms were used in equilibration. Energy minimization and equilibration were both performed with position restrains on heavy atoms. Production MD was then employed with restraints released. Long-range electrostatic interactions were calculated with the smooth particle mesh Ewald method^95,96^ using a 0.12 nm Fourier grid spacing and a 1.2 nm interaction cutoff. Bonds with H atoms were constrained using the *P*-LINCS^49^ algorithm. A 2 fs time step was used. Periodic-boundary conditions were employed in all three spatial dimensions. In total, 36 μs of MD simulation was performed; 18 μs across control systems and 18 μs across POPC-containing systems (3 replicates × 2 μs × 3 oligomer sizes per condition).

**Table 4:** Box Dimensions for Oligomeric Simulations

	Aβ_42_ control	Aβ_42_ + POPC
hexamer	10.1 × 11.5 × 8.9 nm	13.9 × 13.9 × 8.9 nm
octamer	12.7 × 12.4 × 10.3 nm	13.9 × 14.7 × 10.8 nm
decamer	12.2 × 12.2 × 12.2 nm	14.4 × 14.7 × 13.3 nm

### Analysis

Molecular visualization was performed using PyMOL 3.^97^ Data collection was performed using both the GROMACS analysis suite and MDtraj.^98^ All statistical tests were performed using SciPy.^99^ For data sets that satisfied assumptions of equal variance and normality, parametric statistical tests were employed. For normal, multilevel data sets (>2), one-way ANOVA was performed in conjunction with Tukey’s HSD for posthoc comparisons, and for two-group comparisons, *t* tests were used. For data sets that violated assumptions of normality, Kruskal–Wallis was used for multilevel data sets with Dunn’s test for posthoc comparisons, and Mann–Whitney tests were used for two-group comparisons. Statistical tests were performed by selecting random samples from timeseries data to satisfy assumptions of independence. To account for multiple hypothesis testing, *p*-values were adjusted using the False Discovery Rate (FDR) procedure. Significance was defined as p < 0.05 following FDR correction.

### Secondary Structure

Secondary structure calculation was performed using the MDtraj^98^ implementation of the DSSP algorithm.^100^ Simplified DSSP codes were used, where helix, β-strand and coil corresponded to the following: helix (α-helix, 3-helix, and 5/π-helix), β-strand (β-bridge, β-ladder), coil (hydrogen bonded, turn, bend, loops).

### Clustering

RMSD-clustering was performed on protein backbone atoms using the GROMOS method as described by Daura et al.^91^ with a 0.3 nm cutoff. The GROMACS[Bibr ref82] 2020.4 implementation of RMSD clustering was used. Clustering was performed over 200 ns intervals (e.g., 0–200 ns, 200–400 ns...etc.).

### Aggregation State Analysis

For each frame of the simulation, the minimum distance between each Aβ_42_ peptide was calculated. The system was then represented as a NetworkX^101^ graph, where each Aβ_42_ peptide was a node, and edges were defined between nodes if the distance between a given pair of nodes was <0.6 nm. The presence of an N-mer at a given frame was determined by the connectivity of the nodes.

### Radius of Gyration (*R*_g_) and Eccentricity

*R*_g_ was calculated using the GROMACS[Bibr ref82] 2020.4 implementation. Per the GROMACS manual,^102^ *R*_g_ is calculated as in eq 1

1 $$R_{g}={\left(\frac{\sum_{i}{\left|\left|r_{i}\right|\right|}^{2}m_{i}}{\sum_{i}m_{i}}\right)}^{1/2}$$

where *m*_*i*_ is the mass of atom *i*, and *r*_*i*_ is the position of atom *i* with respect to the center of mass of the oligomer.

Eccentricity was derived from moments of inertia around major, middle, and minor inertial axes (*I*_1_, *I*_2_, and *I*_3_, respectively), as calculated by GROMACS.^82^ Given that oligomer structures can be approximated as ellipsoids,^13,103,104^ eccentricity for each simulation frame was calculated as in eq 2

2 $$e=\sqrt{1-\frac{\left(I_{1}+I_{2}-I_{3}\right)}{\left({-I}_{1}+I_{2}+I_{3}\right)}}$$

### Residue Interaction Analysis

The methodology for analysis of intermolecular interactions was performed as described in a previous publication.^65^ Briefly, interaction “frequencies” are calculated, using an interaction cutoff of 0.6 nm, to reduce the dimensionality associated with analyzing residue–residue interactions in multipeptide simulations. Interaction frequency for a given residue pair are weighted based (1) the number of different peptide pairs where the residue–residue interaction occurs, (2) the fraction of frames where the residue–residue interaction occurs, and (3) the mean distance between the residues when within 0.6 nm. For analysis of Aβ_42_ interaction with POPC, Aβ_42_ residue interaction frequency was calculated with respect to various POPC regions (tail, glycerol, phosphate, choline).

Hydrogen bonds were calculated using the MDtraj^98^ implementation of the methodology described by Wernet and Nilsson et al.^105^

### Energy Analysis

Energy analyses were performed using FoldX version 5.0.^106^ Before energy calculations, all PDB files were preprocessed to remove nonprotein components, and each PDB file was first subjected to the RepairPDB command, a recommended standard practice in the FoldX documentation.^72^ This step corrects structural issues such as bad torsion angles and/or steric clashes, ensuring that subsequent residue-level energy calculations are reliable. Subsequently, the SequenceDetail command was employed to compute residue-level energy contributions.^72^

## Supplementary Material



## Notes

Structural clustering analysis and morphologies for Aβ_42_ control and POPC systems; secondary structure probabilities; minimum distance analyses between peptides and between Aβ_42_-POPC molecular regions; aggregation state networks and oligomer formation timelines; radius of gyration and eccentricity distributions; hydrogen bonding patterns between Aβ_42_ and POPC; interpeptide interaction matrices and POPC-induced changes; solvent-accessible surface area versus binding free energy decomposition; water contact analysis; correlation between surface area and binding energy changes (PDF)

## Abbreviations

Aβ: amyloid-β
Aβ40: 40-residue alloform of amyloid-β
Aβ42: 42-residue alloform of amyloid-β
AD: Alzheimer’s disease
ANOVA: analysis of variance
APP: amyloid precursor protein
CMC: critical micelle concentration
DSSP: Dictionary of protein secondary structure
FDR: false discovery rate
HSD: honestly significant difference
MD: molecular dynamics
NMR: nuclear magnetic resonance
*NPT*: constant number of particles, pressure, and temperature ensemble
*NVT*: constant number of particles, volume, and temperature ensemble
P-LINCS: Parallel Linear Constraint Solver
PC: phosphatidylcholine
POPC: 1-palmitoyl-2-oleoyl-*sn*-glycero-3-phosphocholine
*R*_g_: radius of gyration
RMSD: root-mean-square deviation
RMSF: root-mean-square fluctuation
SASA: solvent-accessible surface area
SDS: sodium dodecyl sulfate
TIP3P: Transferable Intermolecular Potential 3-Point water model
Δ*G*: free energy change
