# Supplementary material for: The Influence of POPC as a Coaggregate in Amyloid‑β Oligomer Formation
Source: ACS Chem Neurosci. 2025 Sep 16;16(19):3886–98. doi: 10.1021/acschemneuro.5c00605 (PMC12498404; doi:10.1021/acschemneuro.5c00605)
Supplement: Supplementary file 1 [file cn5c00605_si_001.pdf]

The influence of POPC as a co-aggregate in amyloid-β oligomer formation

Kelsie M. King[†,1], Emma M. Cleveland[†,2], Allison Pennington[3], Sarah Fuccello[3], and Anne M. Brown[*,1,3-5]

[1]Interdisciplinary Program in Genetics, Bioinformatics and Computational Biology, Virginia Polytechnic Institute and State University, Blacksburg, VA.

[2]Department of Systems Biology, Virginia Polytechnic Institute and State University, Blacksburg, VA.

[3]Department of Biochemistry, Virginia Polytechnic Institute and State University, Blacksburg, VA.

[4]Research and Informatics, University Libraries, Virginia Polytechnic Institute and State University, Blacksburg, VA.

[5]Virginia Tech Center for Drug Discovery, Virginia Polytechnic Institute and State University, Blacksburg, VA.

* Correspondence: Anne M. Brown, ambrown7@vt.edu

† Indicates co-first author

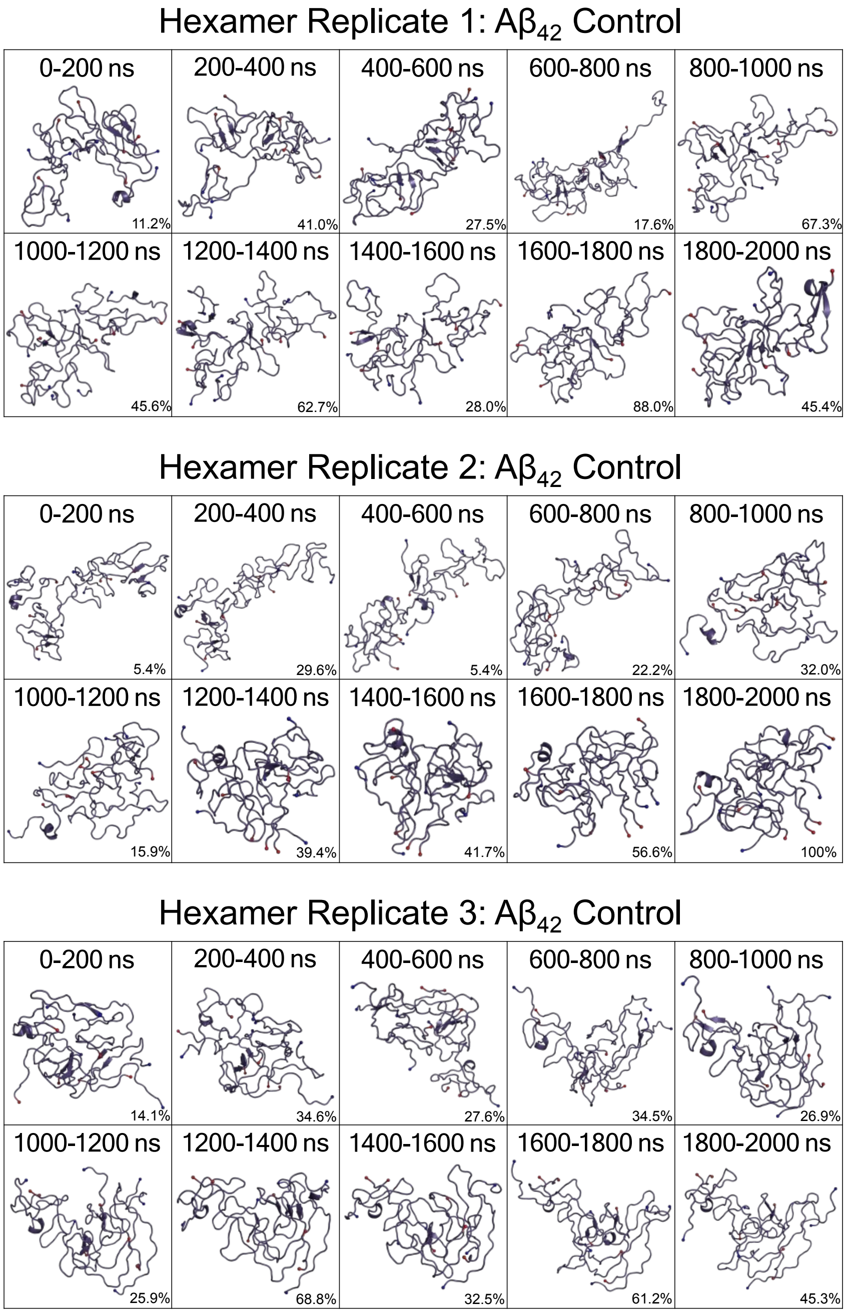

Figure S1. Structural analysis of hexameric Aβ_{42} controls. Dominant structural morphologies derived from root-mean-square deviation (RMSD) clustering analysis for all three replicates of the decamer control. Clustering was conducted using a 0.3 nm cutoff, segmented into intervals spanning 0-200, 200-400, 400-600, 600-800, 800-1000, 1000-1200, 1200-1400, 1400-1600, 1600-1800, and 1800-2000 ns of simulation time. Percentages indicate the percentage of frames the structure represents over the indicated timeframe.

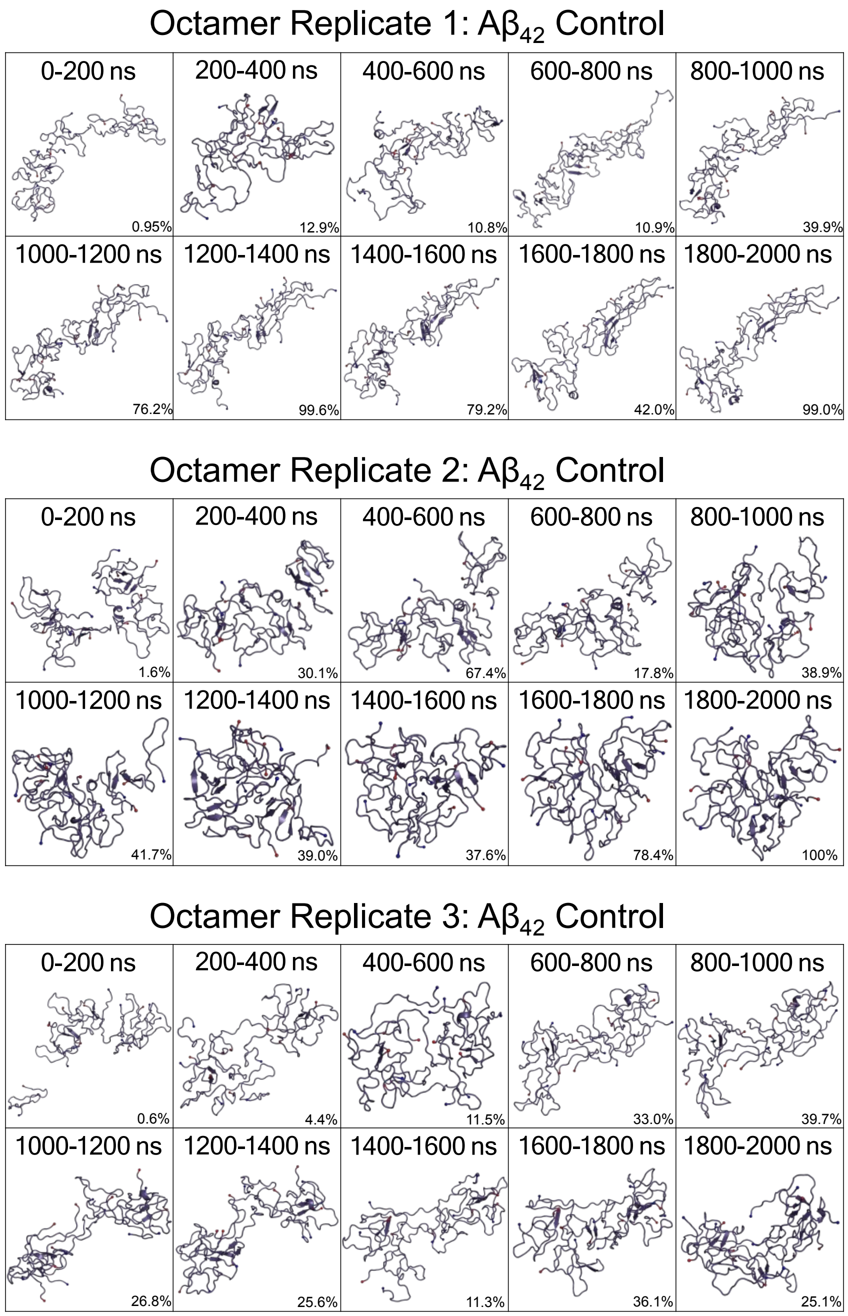

Figure S2. Structural analysis of octameric Aβ_{42} controls. Dominant structural morphologies derived from root-mean-square deviation (RMSD) clustering analysis for all three replicates of the decamer control. Clustering was conducted using a 0.3 nm cutoff, segmented into intervals spanning 0-200, 200-400, 400-600, 600-800, 800-1000, 1000-1200, 1200-1400, 1400-1600, 1600-1800, and 1800-2000 ns of simulation time. Percentages indicate the percentage of frames the structure represents over the indicated timeframe.

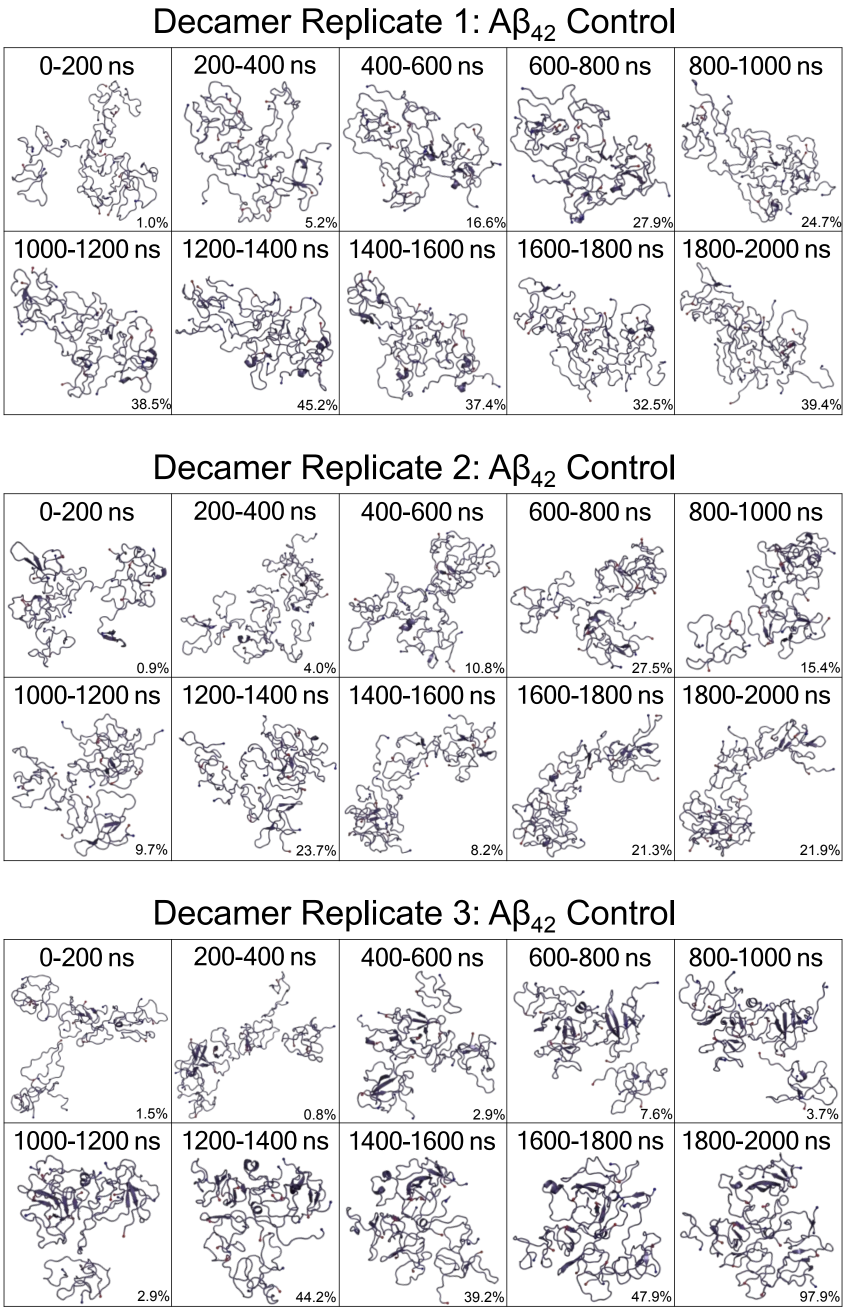

Figure S3. Structural analysis of decameric Aβ_{42} controls. Dominant structural morphologies derived from root-mean-square deviation (RMSD) clustering analysis for all three replicates of the decamer control. Clustering was conducted using a 0.3 nm cutoff, segmented into intervals spanning 0-200, 200-400, 400-600, 600-800, 800-1000, 1000-1200, 1200-1400, 1400-1600, 1600-1800, and 1800-2000 ns of simulation time. Percentages indicate the percentage of frames the structure represents over the indicated timeframe.

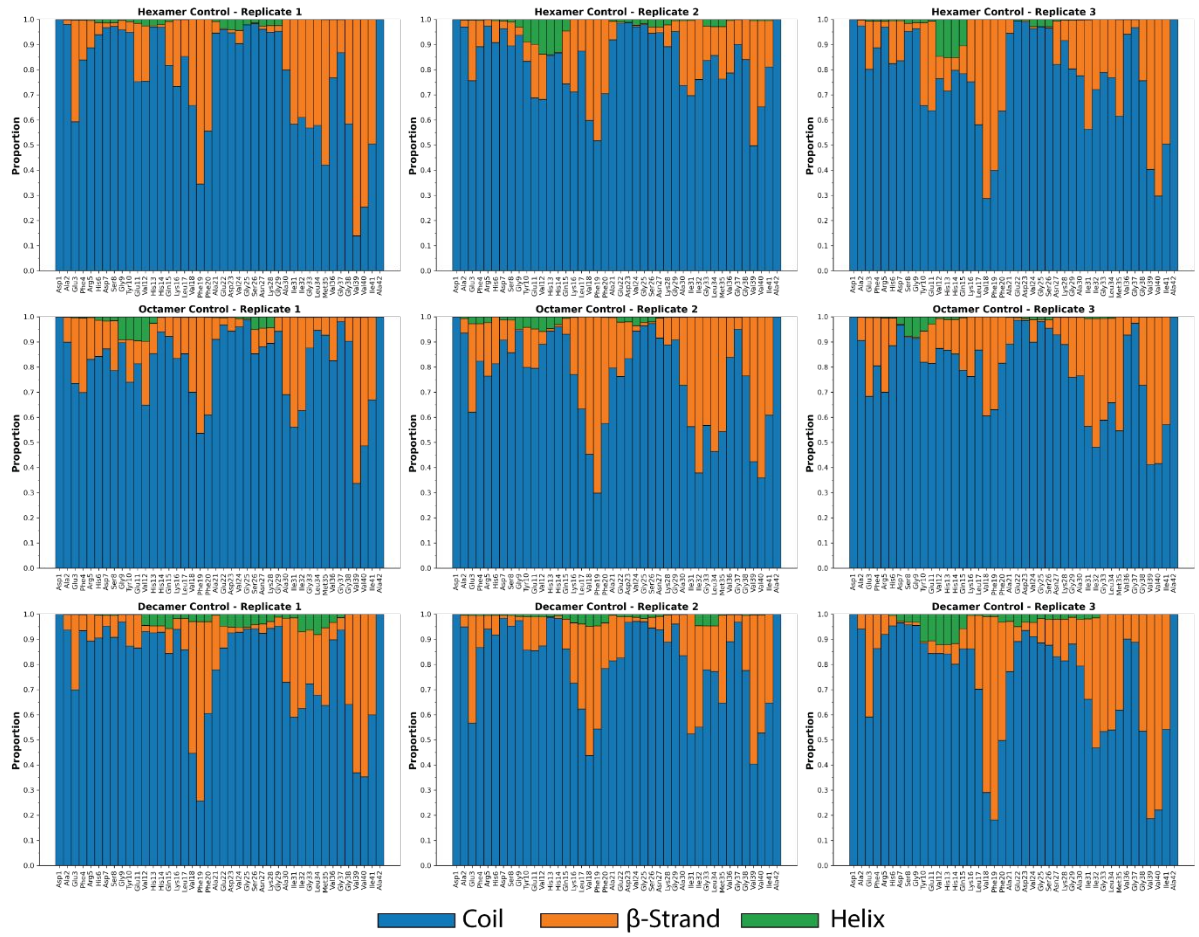

Figure S4. Secondary structure probabilities for Aβ_{42} controls. Probabilities are colored as follows: coil (blue), β-strand (orange), helix (green). Probabilities calculated as percentage of frames a given residue adopts either β-strand, coil, or helical structure throughout the 2 μs simulation period for each replicate.

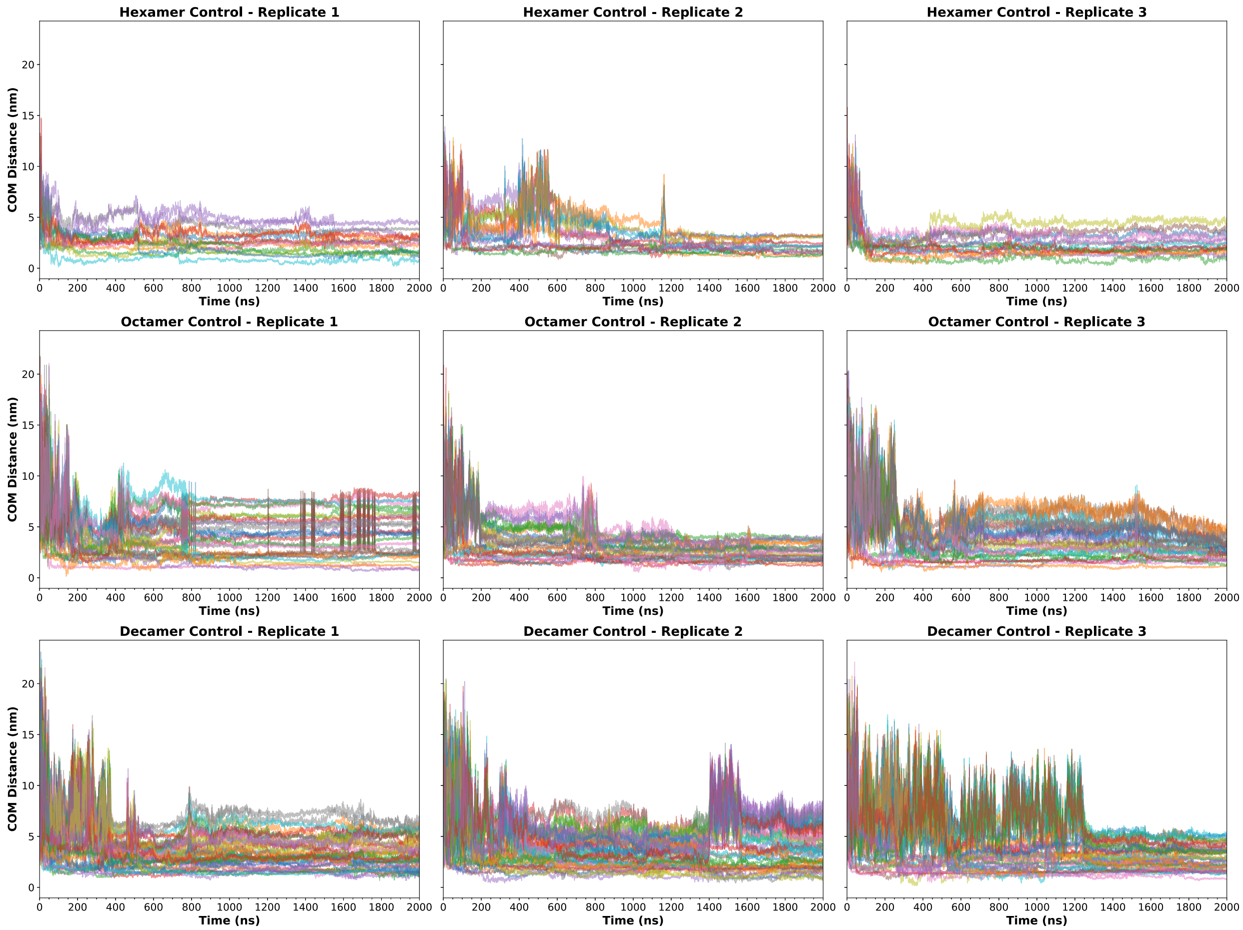

Figure S5. Minimum distance between the center of mass of all peptide pairs for Aβ_{42} controls.

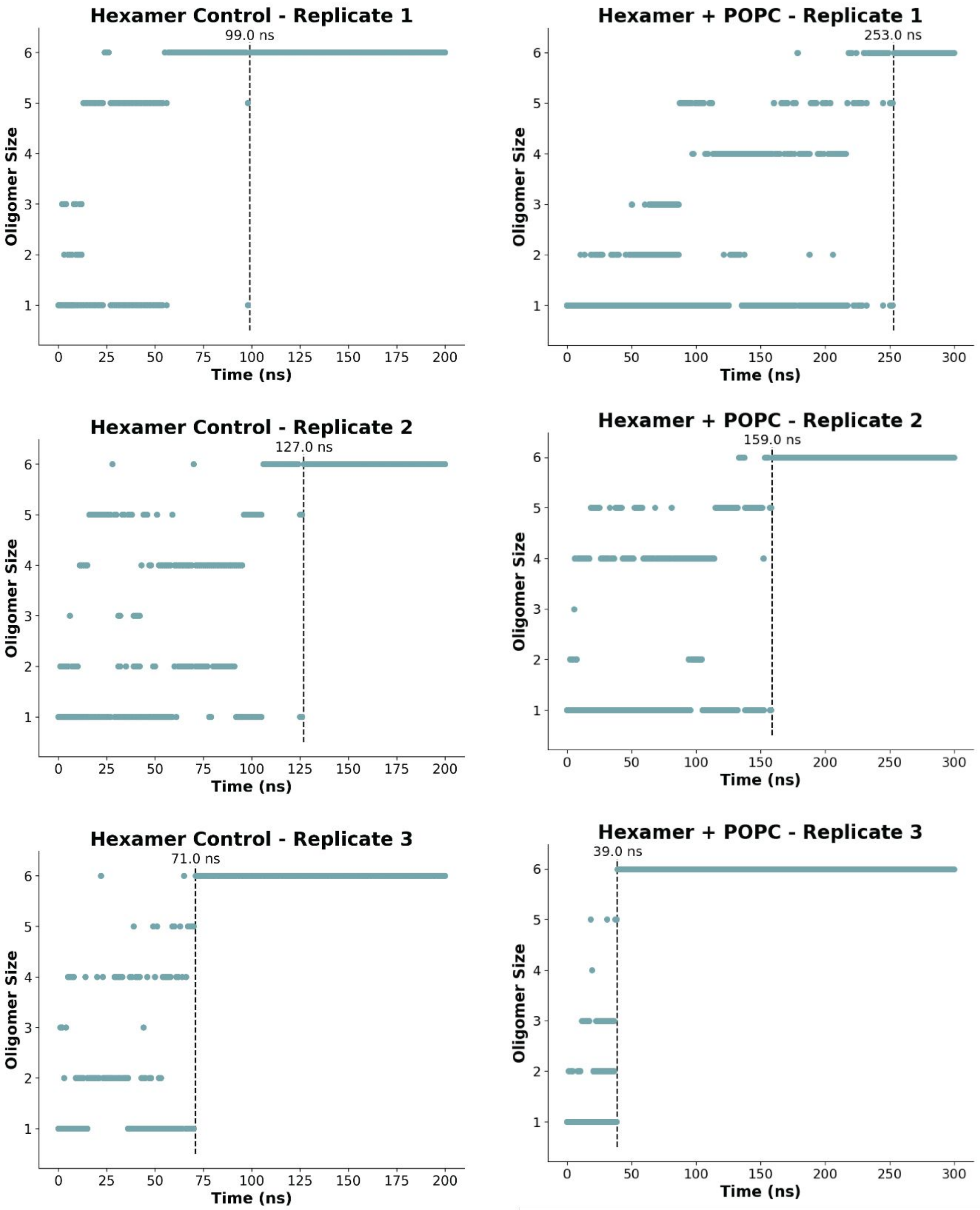

Figure S6. Aggregation state for hexameric Aβ_{42} controls and hexameric Aβ_{42}:POPC. Aggregation state was calculated as the connectivity of a network graph at each frame of simulation. A point on the y-axis corresponds to the existence of an *N*-mer in a given frame. The labeled dashed vertical line corresponds to the time at which a stable higher-order oligomer was formed.

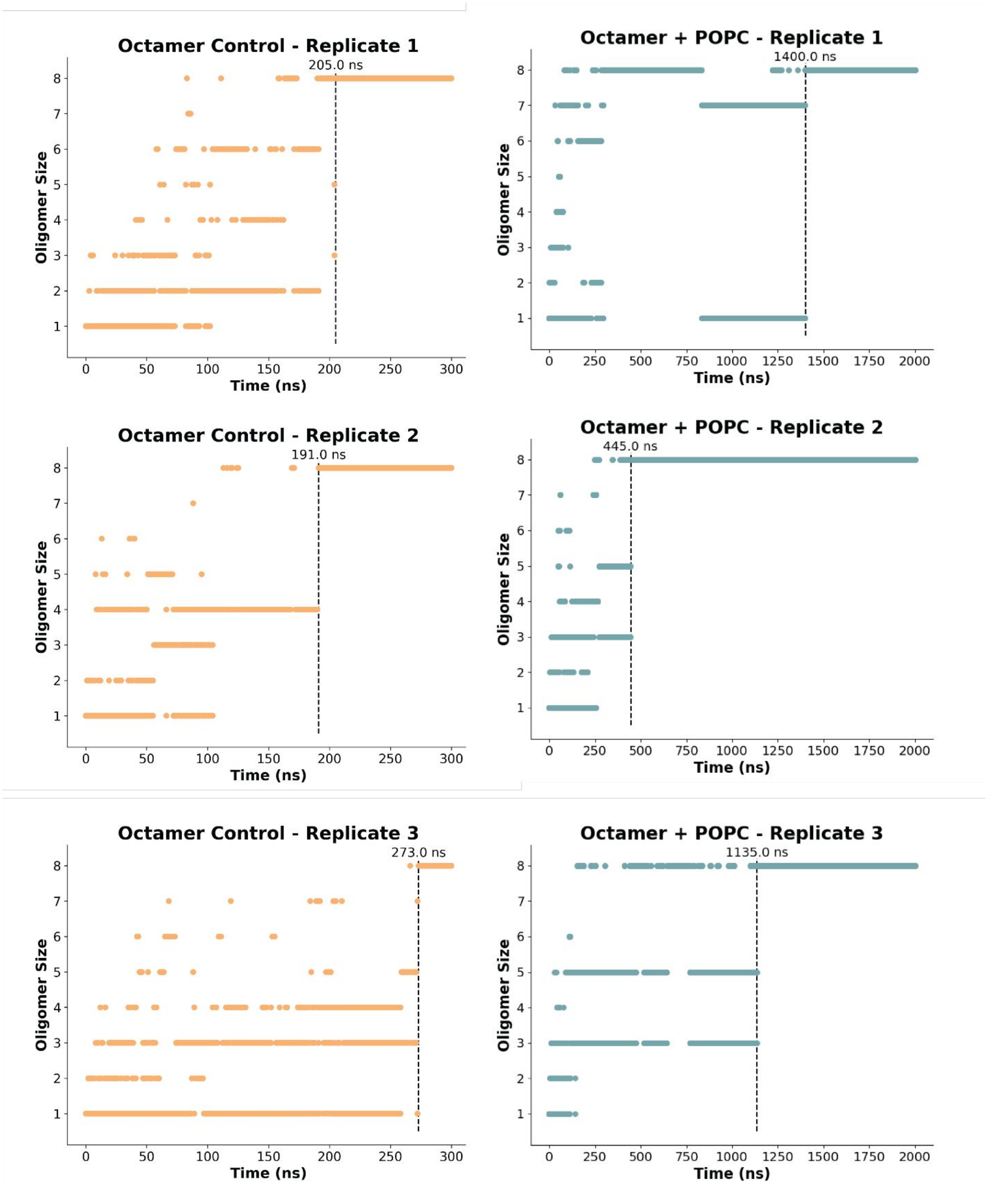

Figure S7. Aggregation state for octameric Aβ_{42} controls and octameric Aβ_{42}:POPC. Aggregation state was calculated as the connectivity of a network graph at each frame of simulation. A point on the y-axis corresponds to the existence of an *N*-mer in a given frame. The labeled dashed vertical line corresponds to the time at which a stable higher-order oligomer was formed.

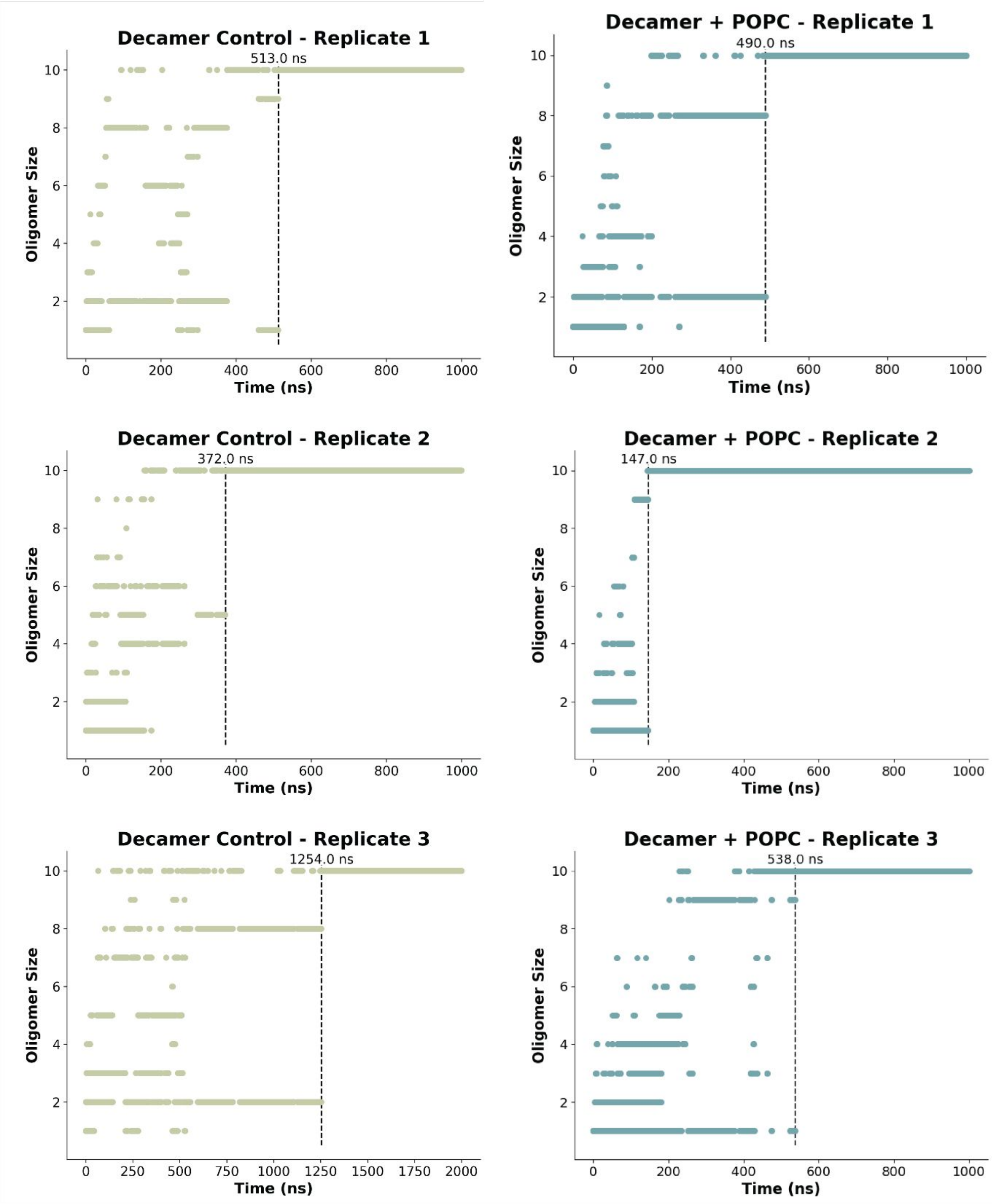

Figure S8. Aggregation state for decameric Aβ_{42} controls and decameric Aβ_{42}:POPC. Aggregation state was calculated as the connectivity of a network graph at each frame of simulation. A point on the y-axis corresponds to the existence of an *N*-mer in a given frame. The labeled dashed vertical line corresponds to the time at which a stable higher-order oligomer was formed.

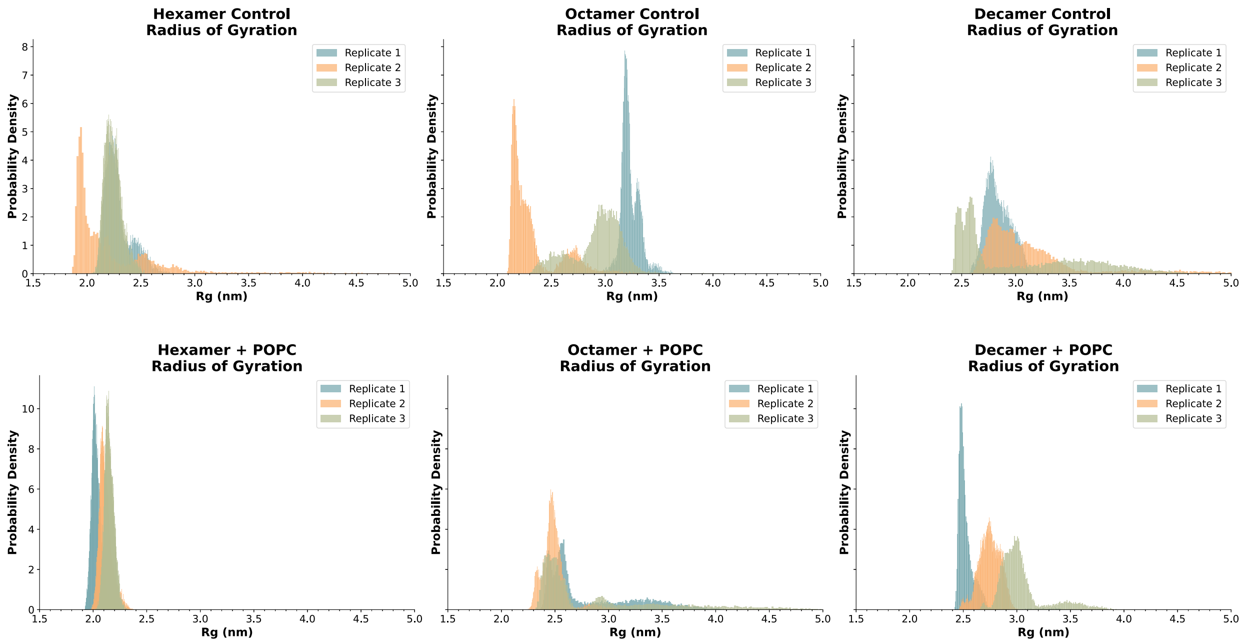

Figure S9. Radius of gyration (Rg) for Aβ_{42} control oligomers and Aβ_{42}:POPC oligomers. Histograms show values from 0.5 – 2 µs of the simulation period, corresponding to the period at which stable (e.g., unchanging in major secondary structure and structural organization) oligomers were formed for all systems (see **Figure S5**).

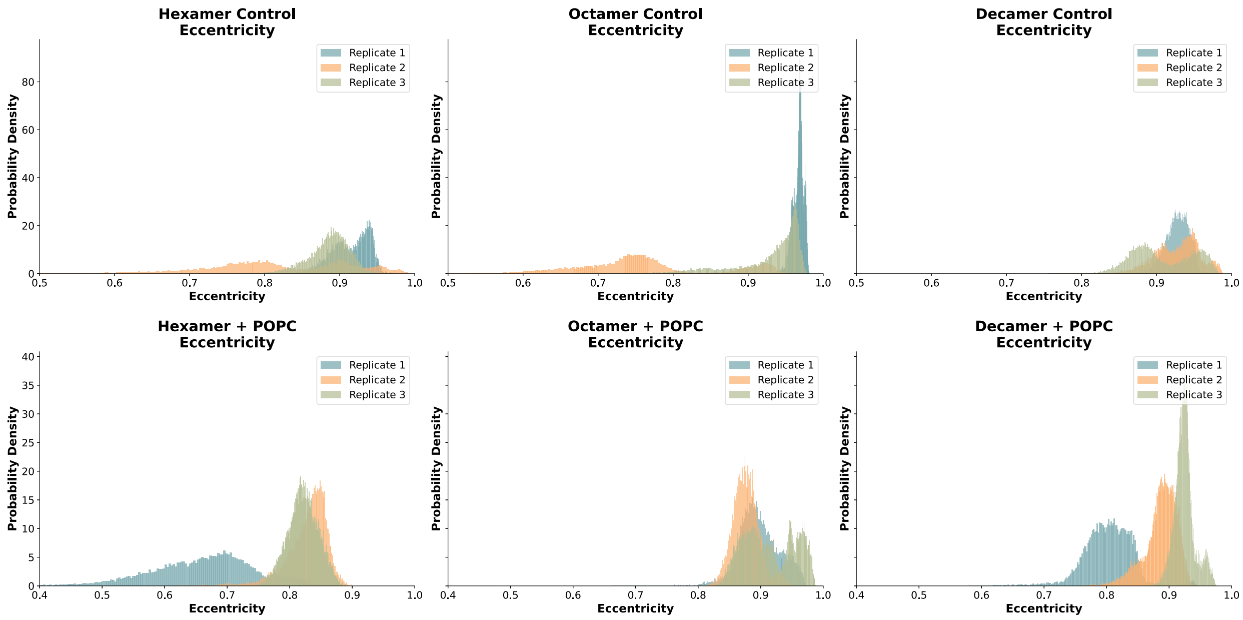

Figure S10. Eccentricity for Aβ$_{42}$ control oligomers and Aβ$_{42}$:POPC oligomers. Histograms show values from 0.5 – 2 μs of the simulation period, corresponding to the period at which stable (e.g., unchanging in major secondary structure and structural organization) oligomers were formed for all systems (see **Figure S5**).

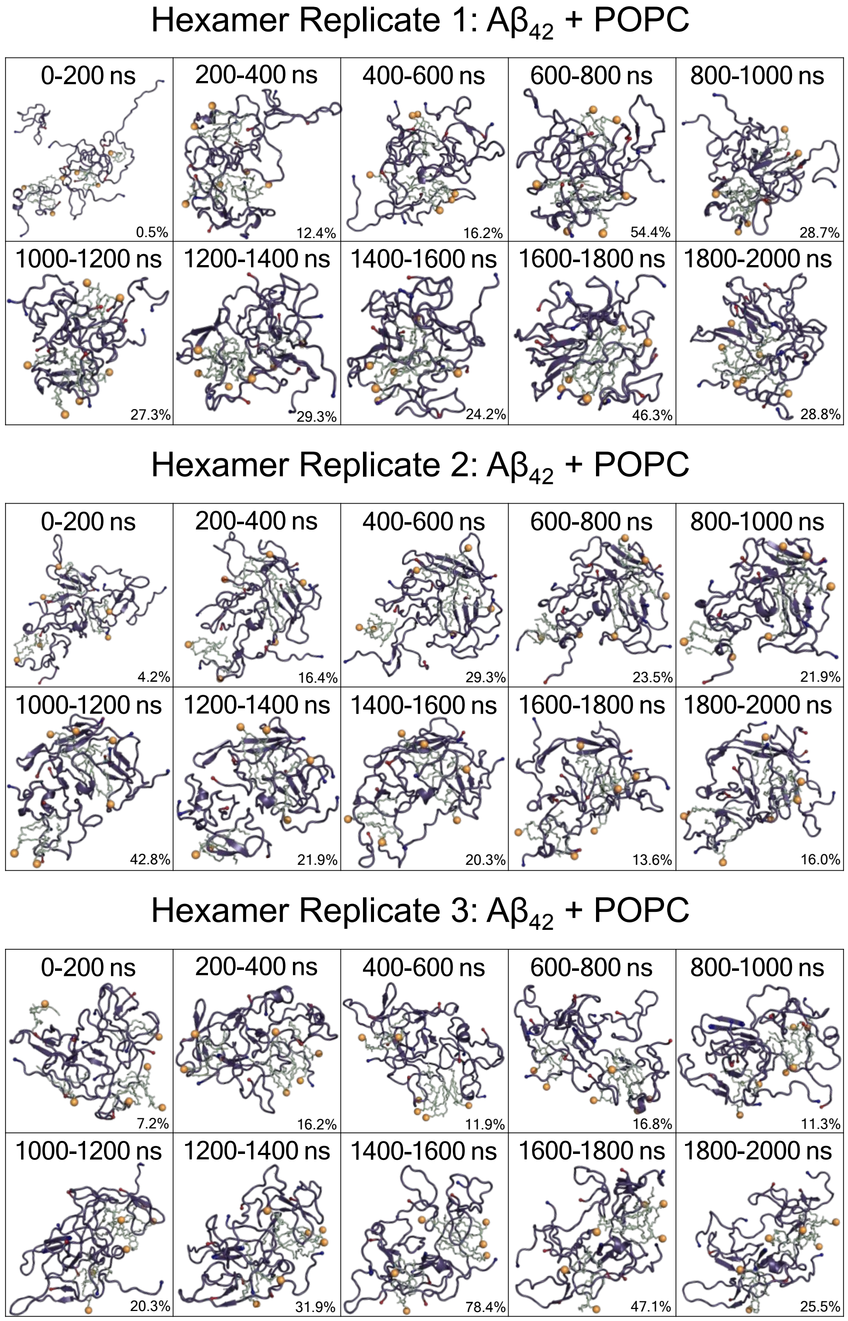

Figure S11. Structural analysis of hexameric Aβ_{42} with POPC. Dominant structure morphologies derived from root-mean-square deviation (RMSD) clustering analysis for all three replicates of the hexameric Aβ_{42} + POPC system. Clustering was conducted using a 0.3 nm cutoff, segmented into intervals spanning 0-200, 200-400, 400-600, 600-800, 800-1000, 1000-1200, 1200-1400, 1400-1600, 1600-1800, and 1800-2000 ns of simulation time. Percentages indicate the percentage of frames the structure represents over the indicated timeframe.

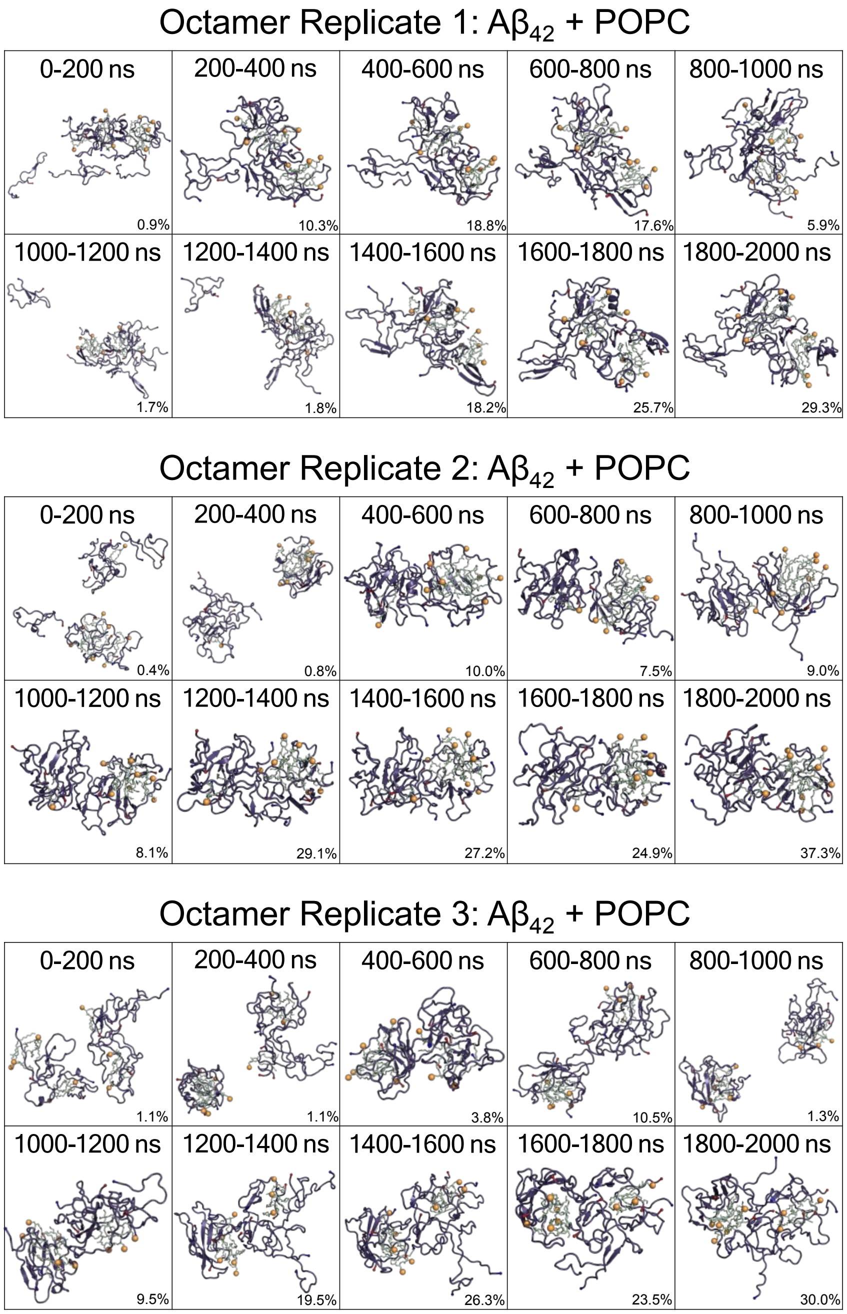

Figure S12. Structural analysis of octameric Aβ_{42} with POPC. Dominant structure morphologies derived from root-mean-square deviation (RMSD) clustering analysis for all three replicates of the hexameric Aβ_{42} + POPC system. Clustering was conducted using a 0.3 nm cutoff, segmented into intervals spanning 0-200, 200-400, 400-600, 600-800, 800-1000, 1000-1200, 1200-1400, 1400-1600, 1600-1800, and 1800-2000 ns of simulation time. Percentages indicate the percentage of frames the structure represents over the indicated timeframe.

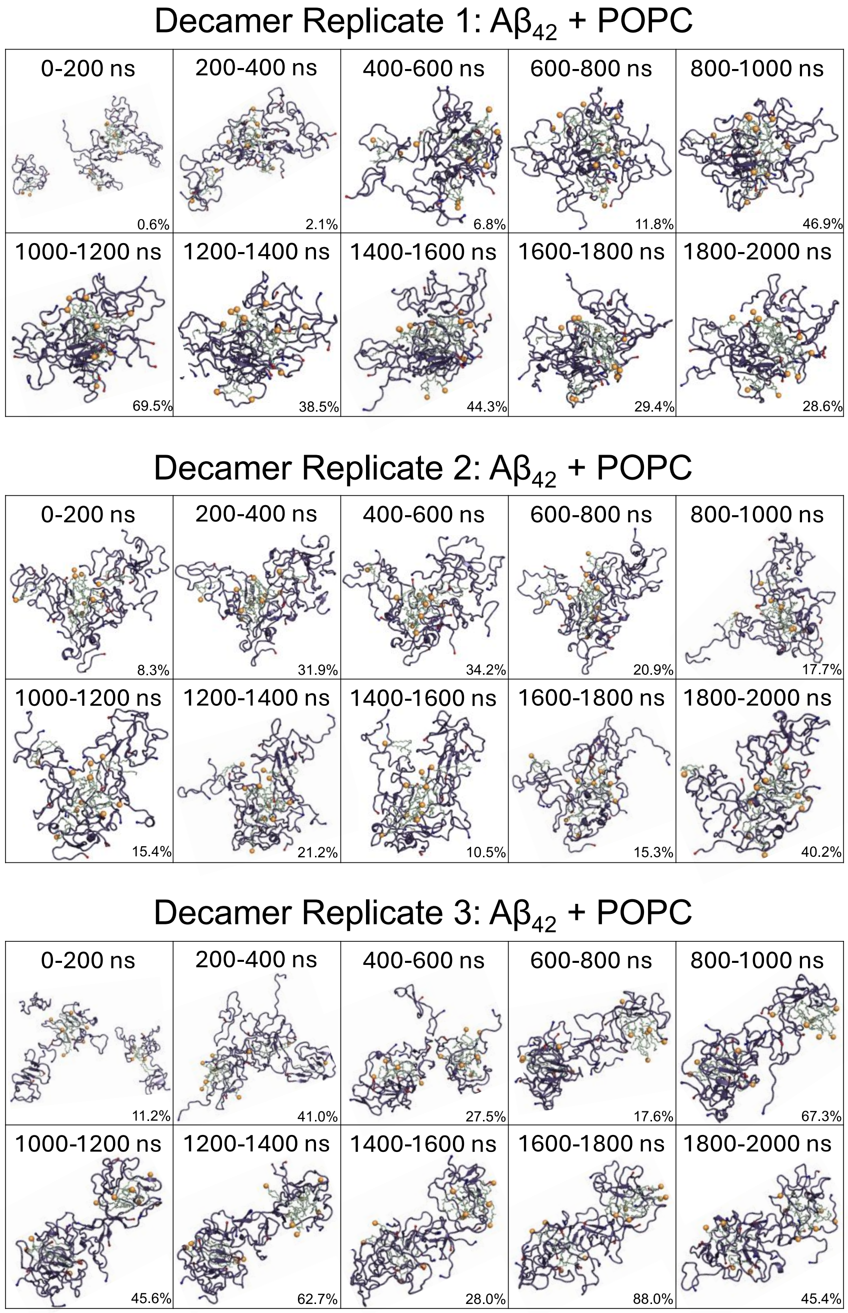

Figure S13. Structural analysis of decameric Aβ_{42} with POPC. Dominant structure morphologies derived from root-mean-square deviation (RMSD) clustering analysis for all three replicates of the hexameric Aβ_{42} + POPC system. Clustering was conducted using a 0.3 nm cutoff, segmented into intervals spanning 0-200, 200-400, 400-600, 600-800, 800-1000, 1000-1200, 1200-1400, 1400-1600, 1600-1800, and 1800-2000 ns of simulation time. Percentages indicate the percentage of frames the structure represents over the indicated timeframe.

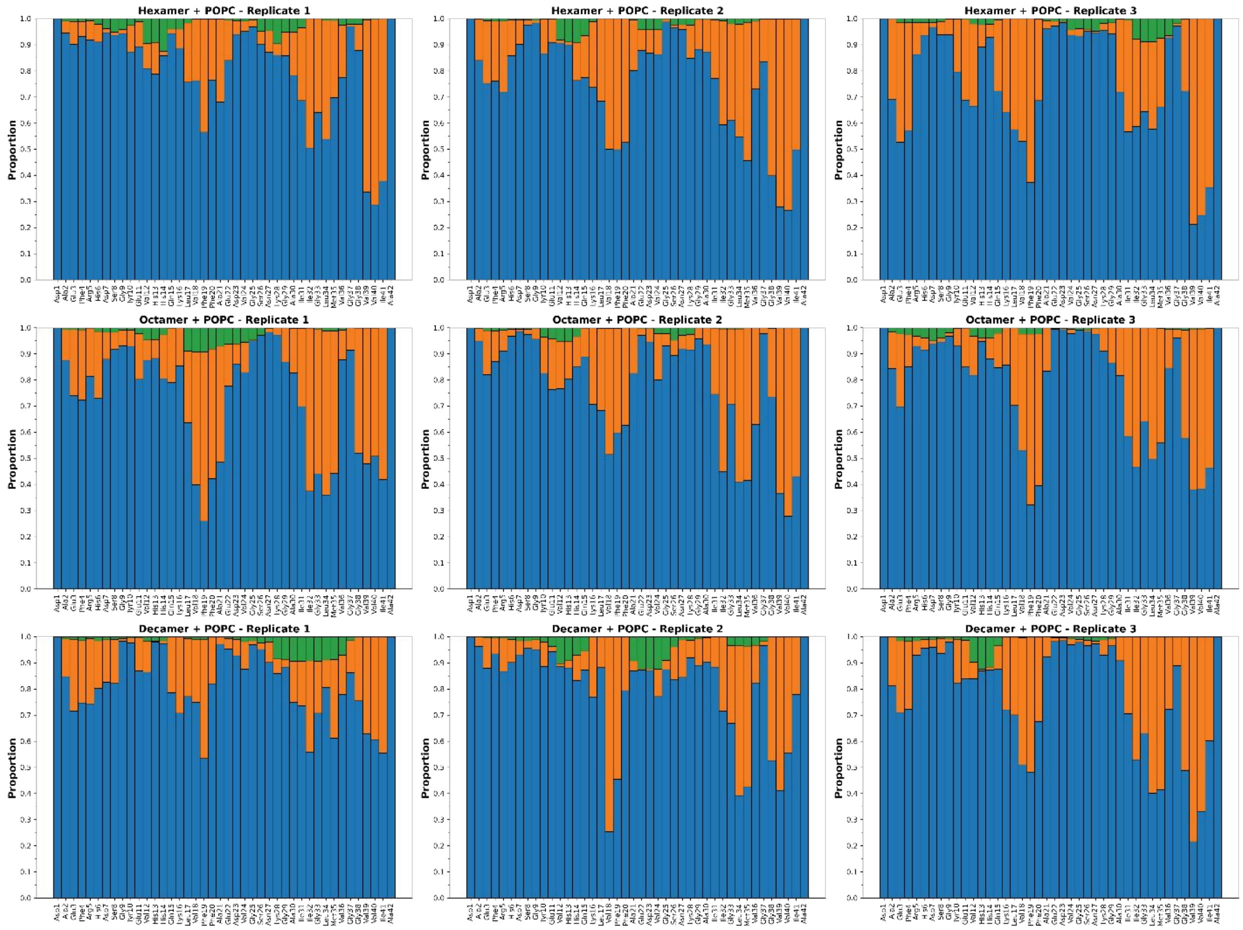

Figure S14. Secondary structure probabilities for Aβ_{42} + POPC. Probabilities are colored as follows: coil (blue), β-strand (orange), helix (green). Probabilities calculated as percentage of frames a given residue adopts either β-strand, coil, or helical structure throughout the 2 μs simulation period for each replicate.

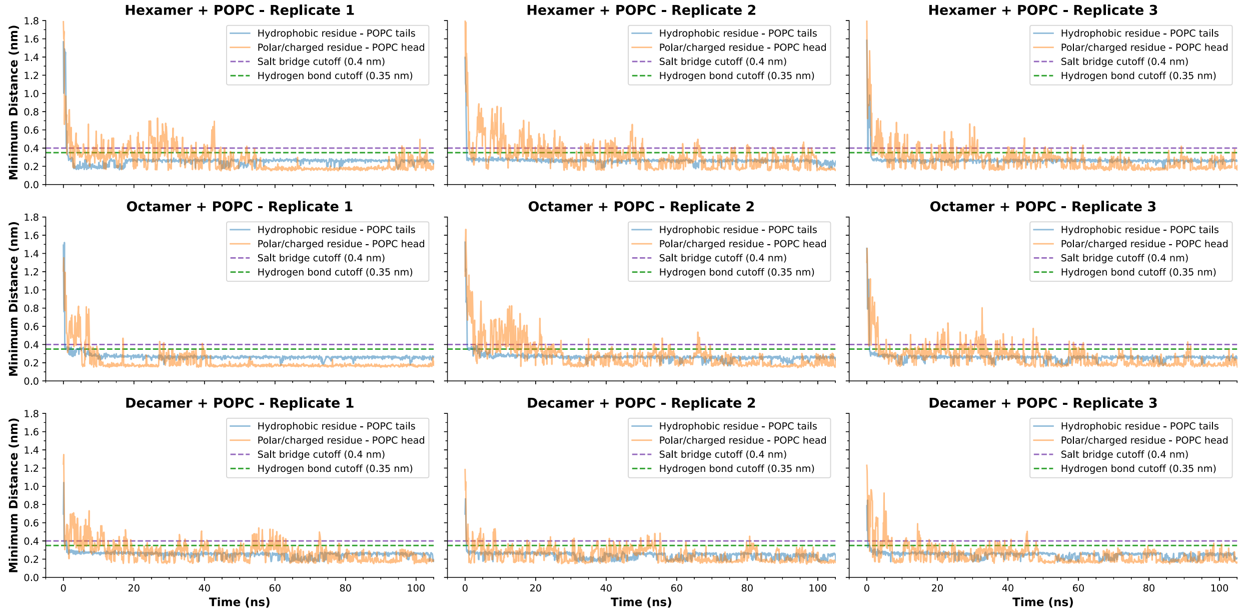

Figure S15. Minimum distance between Aβ$_{42}$ residues and POPC regions in the first 100 ns of simulation. Hydrophobic residues include Ala, Val, Leu, Ile, Met, and Phe residues. Polar/charged residues include Asp, Glu, Arg, Lys, Ser, Thr, Tyr, Asn, and Gln. Minimum distances were calculated to POPC hydrocarbon tails (blue) and the POPC polar headgroup (orange) (see **Figure 4A** for atom designations in POPC tails and POPC head).

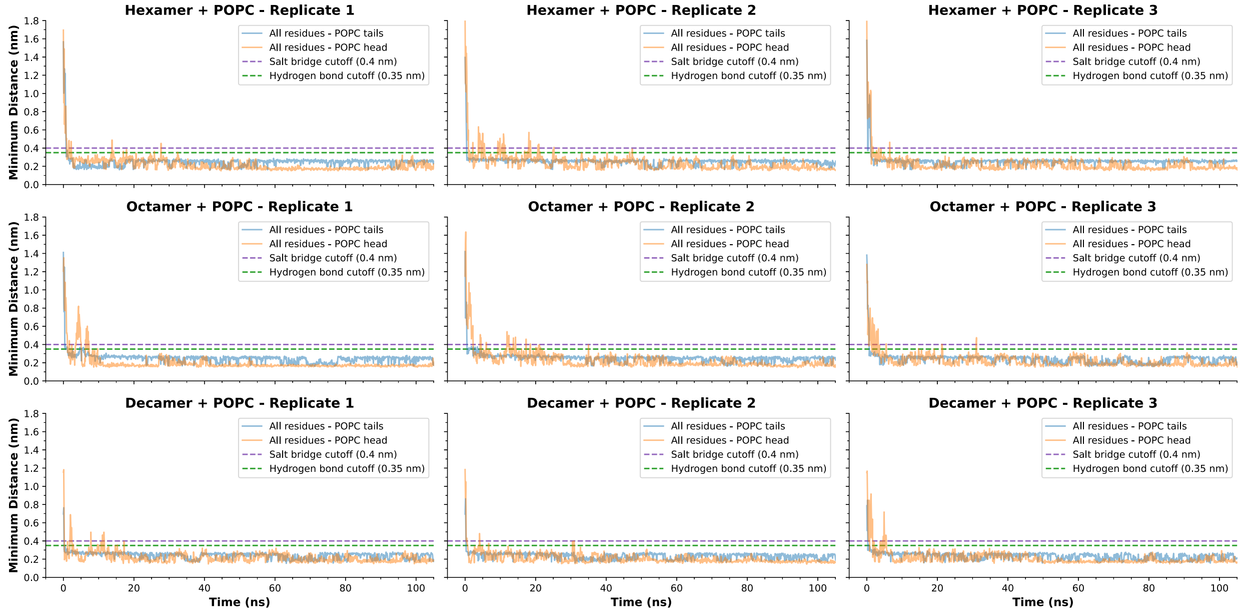

Figure S16. Minimum distance between Aβ$_{42}$ residues and POPC regions in the first 100 ns of simulation. Minimum distances were calculated to POPC hydrocarbon tails (blue) and the POPC polar headgroup (orange) (see **Figure 4A** for atom designations in POPC tails and POPC head).

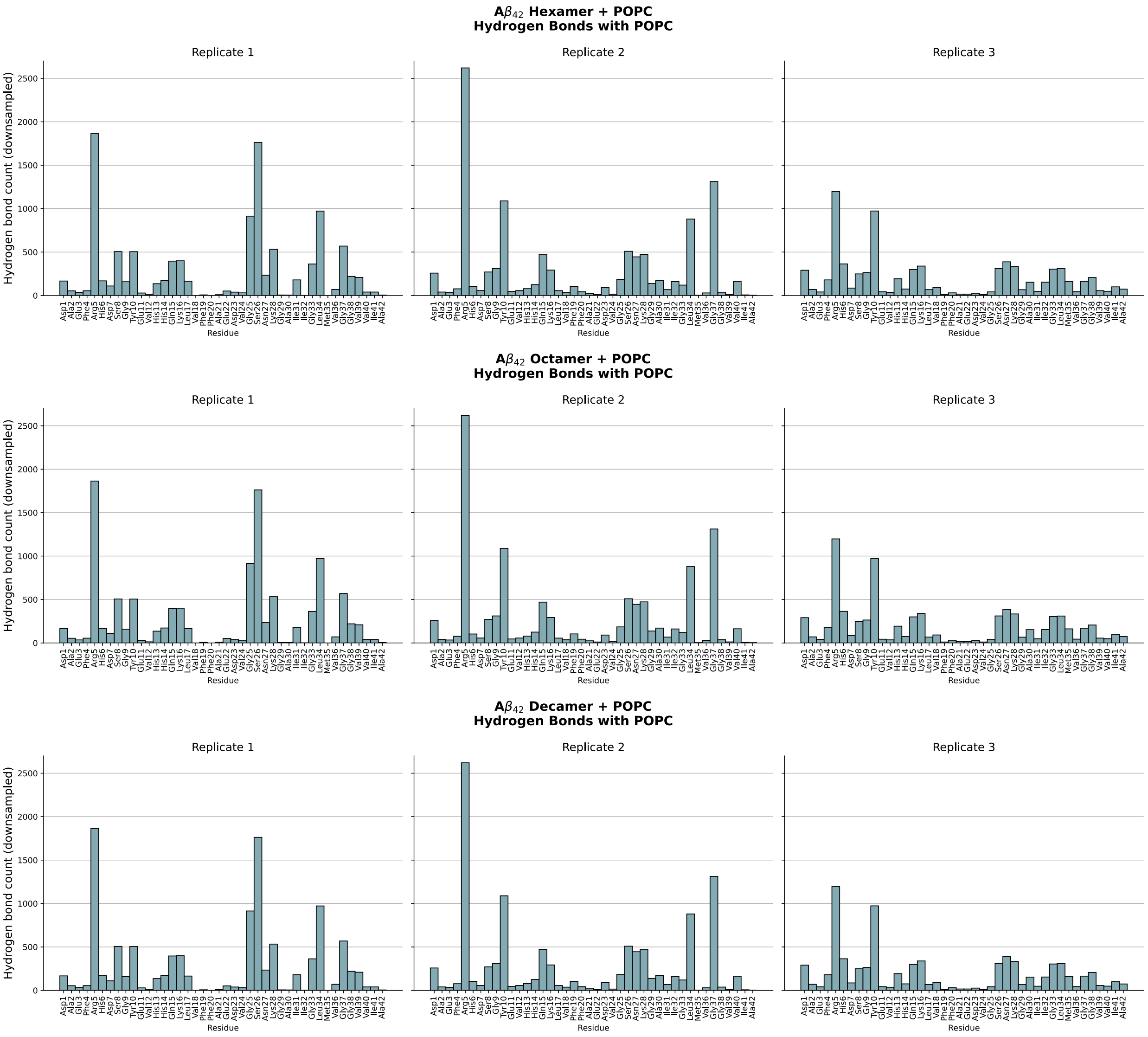

Figure S17. Hydrogen bond counts between Aβ_{42} residues and POPC. Counts taken from a downsampled dataset trajectory (every 10 ps) to increase computational efficiency. The counts represent the number of hydrogen bonds between a residue and POPC over the entire downsampled trajectory, and summed across chains.

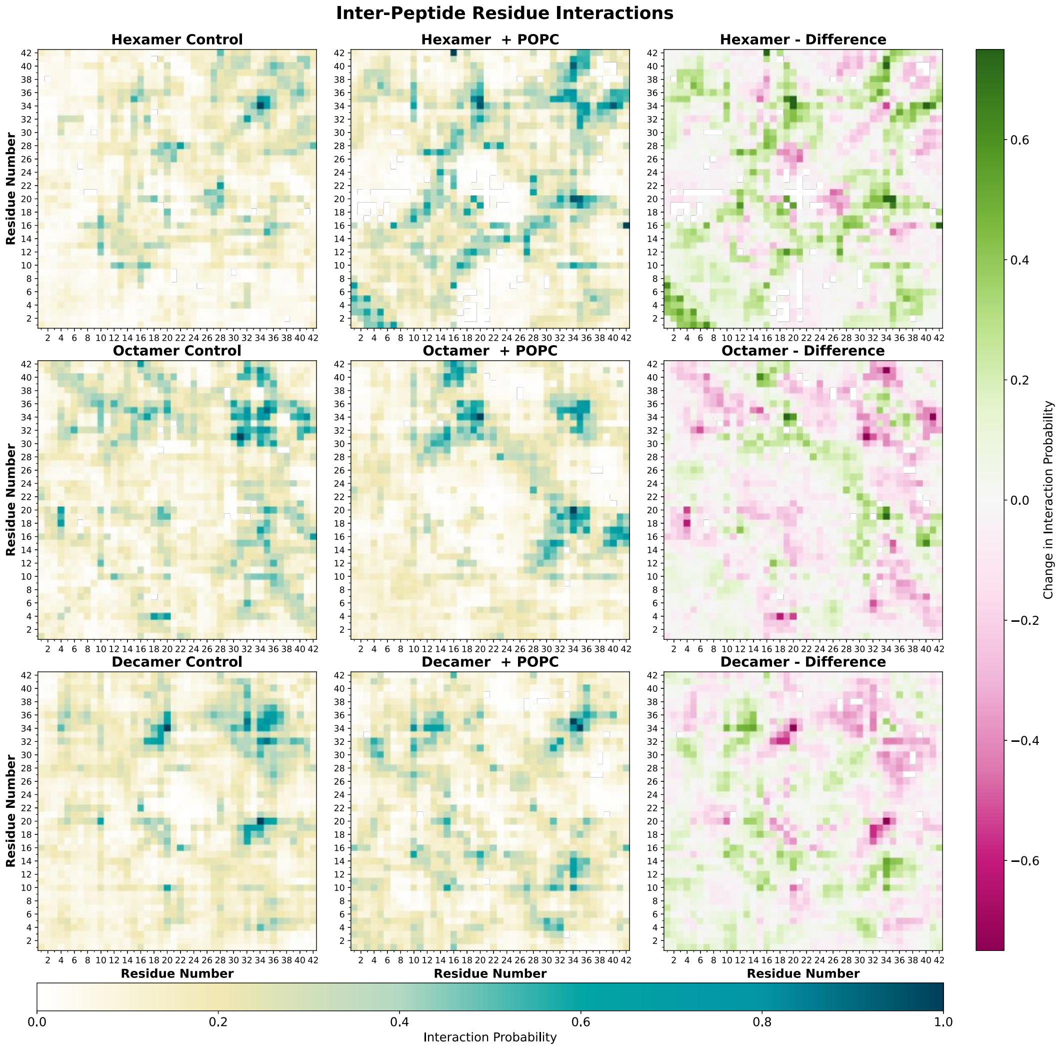

Figure S18. Composite inter-peptide residue interaction probabilities for Aβ$_{42}$ controls, Aβ$_{42}$ with POPC, and the change in interaction propensity between control systems and POPC systems. (*Left & middle columns*) Heatmaps represent a composite of all three replicates over the 2 μs simulation period. (*Right column)* Difference in composite probability. The change is calculated as middle column (oligomer + POPC) subtracted from the leftmost column (oligomer control). White indicates no change. Green indicates increased interaction between residue pairs in Aβ42:POPC oligomers with respect to the control. Red/pink indicates decreased interaction between residue pairs in Aβ42:POPC oligomers with respect to the control.

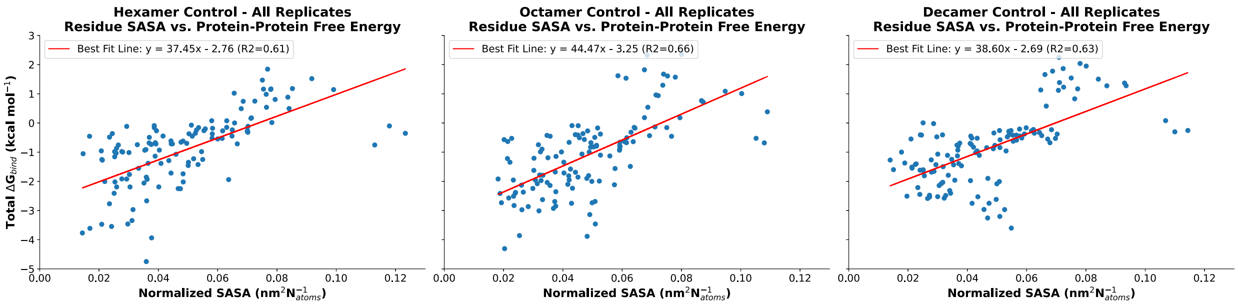

Figure S19. Normalized solvent-accessible surface area (SASA) *vs.* total free energy of binding decomposition (ΔG_{bind}) for control Aβ_{42} hexamer, octamer, and decamer systems. SASA per-residue is normalized by dividing the SASA by the number of atoms in the residue. The total free energy decomposition is the sum of calculated free energy components, including van der Waals, electrostatic, polar solvation, non-polar solvation, and internal energies.

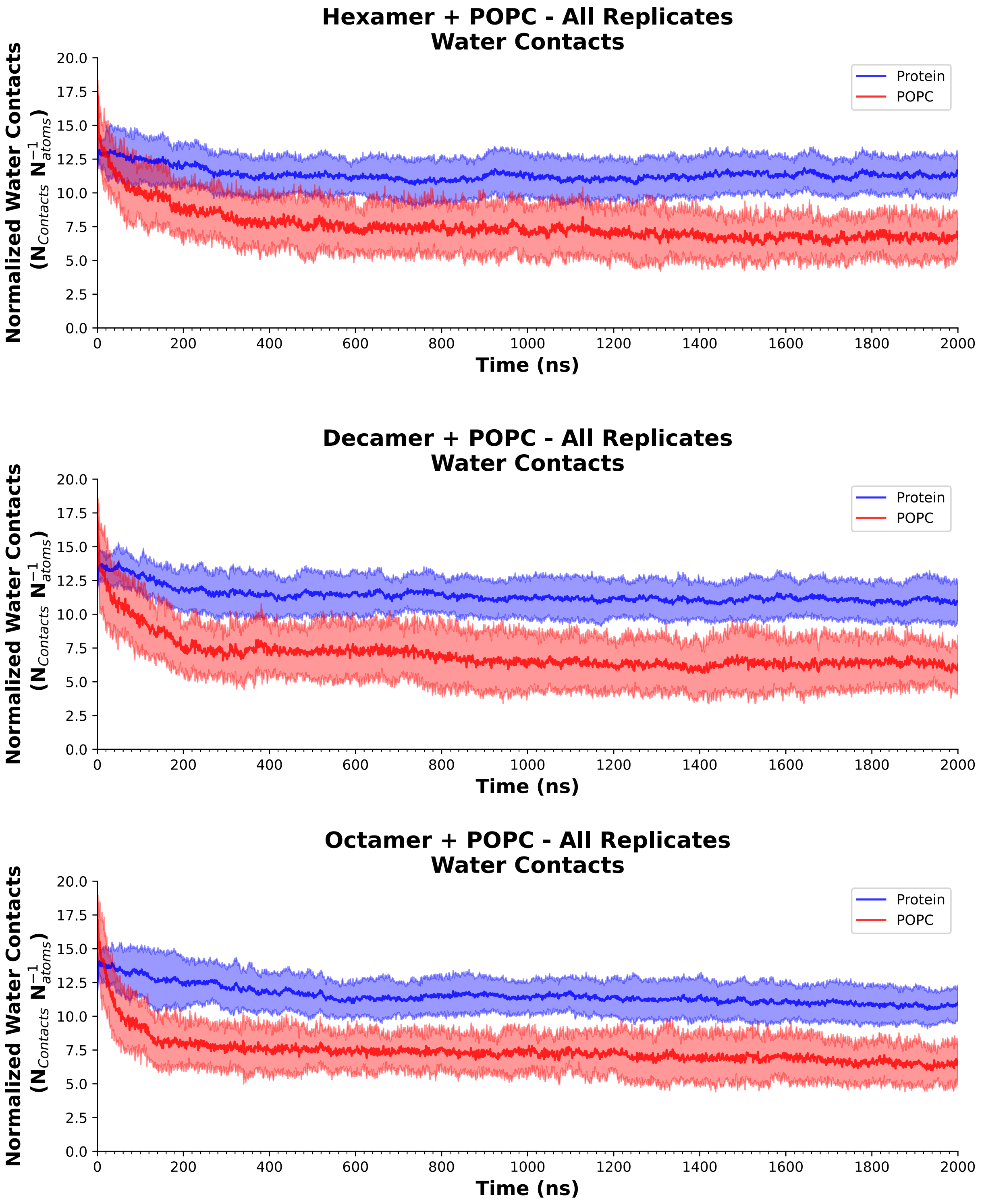

Figure S20. Average number of contacts with water for Aβ$_{42}$ peptides and POPC lipids. The number of contacts with water were normalized by the number of atoms in each chain to adjust for molecule size. Counts represent the average for all chains and replicates at each time point of the simulation. The standard deviation is shown as a translucent fill.

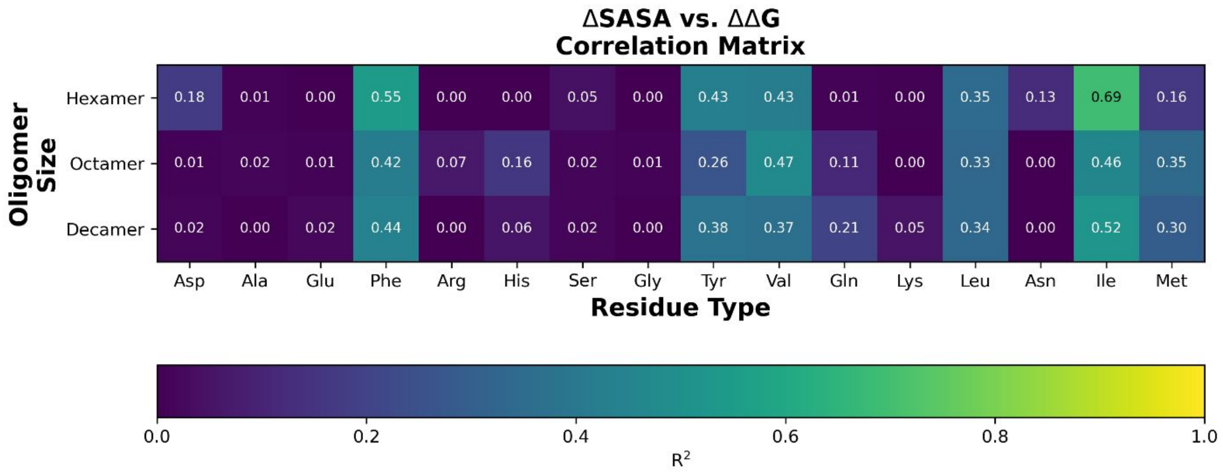

Figure S21. Correlation matrix between ΔSASA and ΔΔG by residue type. R^2 was calculated by correlating residue ΔΔG ($\Delta G_{POPC} - \Delta G_{Control}$) with residue ΔSASA ($SASA_{POPC} - SASA_{Control}$) in a linear regression model.
